# Myosin 1g Contributes to CD44 Adhesion Protein and Lipid Rafts Recycling and Controls CD44 Capping and Cell Migration in B Lymphocytes

**DOI:** 10.3389/fimmu.2017.01731

**Published:** 2017-12-11

**Authors:** Orestes López-Ortega, Leopoldo Santos-Argumedo

**Affiliations:** ^1^Departamento de Biomedicina Molecular, Centro de Investigación y de Estudios Avanzados del Instituto Politécnico Nacional, Ciudad de México, México

**Keywords:** B lymphocyte, recycling, cytoskeleton, adhesion molecules, myosin

## Abstract

Cell migration and adhesion are critical for immune system function and involve many proteins, which must be continuously transported and recycled in the cell. Recycling of adhesion molecules requires the participation of several proteins, including actin, tubulin, and GTPases, and of membrane components such as sphingolipids and cholesterol. However, roles of actin motor proteins in adhesion molecule recycling are poorly understood. In this study, we identified myosin 1g as one of the important motor proteins that drives recycling of the adhesion protein CD44 in B lymphocytes. We demonstrate that the lack of Myo1g decreases the cell-surface levels of CD44 and of the lipid raft surrogate GM1. In cells depleted of Myo1g, the recycling of CD44 was delayed, the delay seems to be caused at the level of formation of recycling complex and entry into recycling endosomes. Moreover, a defective lipid raft recycling in Myo1g-deficient cells had an impact both on the capping of CD44 and on cell migration. Both processes required the transportation of lipid rafts to the cell surface to deliver signaling components. Furthermore, the extramembrane was essential for cell expansion and remodeling of the plasma membrane topology. Therefore, Myo1g is important during the recycling of lipid rafts to the membrane and to the accompanied proteins that regulate plasma membrane plasticity. Thus, Myosin 1g contributes to cell adhesion and cell migration through CD44 recycling in B lymphocytes.

## Introduction

The ability of cells to generate polarized distributions of several molecules enables numerous biological process including asymmetric cell division, cell motility, and formation of the immunological synapse. This polarization comprises signaling molecules, GTPases, adhesion molecules, the MHC complex, cytoskeleton-binding proteins, and myosins, among others (1).

Cell migration and cell adhesion are critical processes in cells of the immune system. For example, during inflammation, leukocytes are recruited by activated endothelial cells followed by transmigration, and their establishment in the injured tissue (1). These phenomena require cell adhesion and cell migration. Over these serial processes, the cell undergoes multiple attachments and detachments to endothelial cells (1). These processes are regulated by several proteins, which include adhesion molecules such as integrins and CD44. During migration, the adhesion proteins function as traction points that drive the formation and stabilization of membrane projections, thus allowing the movement of cells (2).

During migration, these molecules constantly shuttle between different cell compartments and the plasma membrane; this process is called recycling. This recycling process requires the maintenance of a constant state of equilibrium within the internal cellular membranes and the plasma membrane (3). The vesicular traffic is a complex mechanism involving membrane compartments, which differ in their lipid and protein composition, thereby providing specificity in their content and functions (4). Various proteins, involved in vesicular transport have been described, including clathrin, caveolin, dynamin, GTPases, synaptotagmin, actin, myosins, tubulin, and dyneins (5).

Several traffic pathways have been described, such as, clathrin or caveolin-dependent mechanisms. For example, adhesion molecules migrate to small dynamic subdomains that can be stabilized to form larger specialized caveolin-dependent microdomains (3). These sphingolipid- and cholesterol-enriched lipid microdomains (lipid rafts) recruit several protein components and compartmentalize cellular processes associated with cell signaling and membrane trafficking (4). Furthermore, it has recently been shown that when a cell detaches from a surface, these lipid rafts are rapidly internalized (5, 6). After endocytosis, these membranes and associated rafts are delivered into a caveolin-, actin-, and microtubule-dependent pathway to a perinuclear recycling endosome or Golgi apparatus, which serves as a membrane storage compartment (7). In recent years, it has been demonstrated that the caveolin-dependent pathway participates in the endocytosis of integrins (8, 9) such as LFA-1 (10, 11), and molecules associated with lipid rafts, such as MHC-II (12), CD55, CD59 (13), and CD44 (14).

CD44 is a class I transmembrane glycoprotein, and one of its ligands is hyaluronic acid (HA), a component of the extracellular matrix (15). CD44 interacts with the actin-cytoskeleton *via* molecules from the Ezrin, Radixin, and Moesin family (16, 17). CD44 is involved in many cellular processes such as differentiation and motility of normal cells or cell migration and metastasis in different types of cancer cells (18, 19). Previous studies have shown that CD44-deficient mice have abnormalities in myeloid-progenitor migration, bone marrow colonization (20), and homing of lymphocytes to lymph nodes or the thymus (21). CD44 is located at the leading edge and lamellipodia of several cell types *in vitro* (22).

CD44 and integrins mediate re-adhesion of the cell during cell spreading, cell adhesion, and cell migration. This attachment and detachment is controlled by the endocytosis and exocytosis of proteins resident in lipid rafts (7). This adhesion and re-adhesion cycle that delivers the signaling components and the extra membrane required for cell surface expansion and remodeling of plasma membrane are essential for cell migration (4, 23). This process is known to involve small GTPases, but the specific actin-motor proteins (myosins) required to deliver vesicles containing these lipid rafts to the plasma membrane have not yet been identified.

Myosins are a family of proteins characterized by their ability to bind to filamentous actin. These proteins possess ATPase activity, which promotes the hydrolysis of ATP coupled with conformational changes. These changes allow movement along the microfilament. As a result, they are called motor proteins (24, 25). Myosins are the primary microfilament-associated motor proteins. They represent likely candidates to mediate the recycling of adhesion molecules. Myosins occur as monomeric or dimeric motors with a diverse range of cellular roles, such as transporters, anchors, or for tension maintenance (26). Class I myosins have eight members (Myo1a-Myo1h) (27–29). Myosin1g (Myo1g) is a monomeric class I myosin with a single N-terminal catalytic motor (head) domain, a regulatory neck region that contains IQ-motifs for calmodulin binding, and a C-terminal tail, which directly associates, through a putative pleckstrin homology domain, with phosphatidylinositol 3,4-bisphosphate (30) and phosphatidylinositol 3,4,5-triphosphate (31) in membranes.

Myo1g is expressed in hematopoietic cells and has been shown to localize to the plasma membrane (30). It is important to bind the plasma membrane to the actin-cytoskeleton in lymphocytes (32). It also plays a role in the phagocytosis of opsonized-microbeads in macrophages (31) and is involved in cell spreading and cell adhesion in B lymphocytes (33). Furthermore, Myo1g is present in several types of vesicles such as endosomes (34) and exosomes in T (35) and B-lymphocytes (36), as determined by mass spectrometry.

Therefore, the objective of this study was to characterize the cellular functions of Myo1g in B lymphocytes. We demonstrate that Myo1g is a motor protein that is crucial for the cellular distribution and trafficking of CD44, associated with lipid rafts. Myo1g participates in the recycling of vesicles enriched in GPI-anchored proteins. Depletion of Myo1g leads to a loss of CD44 and lipid rafts from the plasma membrane, which suggests a role for Myo1g in the exocytosis of lipid raft membranes and protein associates from an intracellular recycling compartment. These results reveal a novel molecular function, important for cell capping, by which Myo1g mediates lipid raft exocytosis to dynamic sites of the plasma membrane.

## Experimental Procedures

### Mice and Reagents

Female C57BL/6J WT or C57BL/6J Myo1g-deficient (Myo1g^−/−^) mice (8–12 weeks of age) (33) were used in all experiments. The mice were produced at the Centro de Investigación y de Estudios Avanzados (Mexico City, Mexico) animal facility. The Animal Care and Use Committee of Centro de Investigación y de Estudios Avanzados approved all experiments. Antibodies and reagents used in this study are as follows: purified rabbit polyclonal immunoglobulin (Ig)G α-Myo1g (30), rabbit α-Myo1g (Santa Cruz Biotechnology, Dallas, TX, USA), goat α-Myo1g (Santa Cruz Biotechnology), rat monoclonal antibodies (mAb) NIM-R1 (α-Thy-1) and NIM-R8 (α-CD44) (37), biotin-labeled mAb α-CD44 (clone IM7) (BD Pharmingen, San Diego, CA, USA), α-rat-PE (BD Pharmingen), purified α-goat-PE, α-rabbit IgG-Alexa488 (Molecular Probes, Eugene, OR, USA), α-rabbit IgG-FITC (Jackson ImmunoResearch, West Grove, PA, USA), α-rabbit IgG-Cy3 (Jackson ImmunoResearch), Streptavidin-Cy3 (Invitrogen, Frederick, MD, USA), TRITC-phalloidin (Molecular Probes), FITC-phalloidin (Sigma Chemical Co, St. Louis, MO, USA), Streptavidin-APC (BD), Pacific Blue α-B220 (BD Pharmingen) α-IgM (Zymed Laboratories, San Francisco, CA, USA), α-Rab5 (Santa Cruz Biotechnology), α-CDC42 (Santa Cruz Biotechnology), α-CDC42-GTP (New East Biosciences, Brownsville, TX, USA), α-RhoA (Santa Cruz Biotechnology), α-RhoA-GTP (New East Biosciences), α-Actin (Santa Cruz Biotechnology), mAb α-CD44 (clone KM201, Abcam Cambridge, UK), α-Ezrin (Santa Cruz Biotechnology), α-Caveolin (Santa Cruz Biotechnology), purified goat polyclonal IgG α-Rab11 (38), biotinylated cholera toxin subunit B (Life Technologies, Grand Island, NY), Streptavidin-FITC (Sigma), α-CD44-PE (BioLegend, San Diego, CA, USA), and α-B220-VB421 (BioLegend), Primaquine (Sigma), Lanosterol (Sigma), Cholesterol (Sigma), Golgi-stop (Becton Dickinson, East Rutherford, NJ, USA).

### B Lymphocyte Isolation, Activation, and Staining

Mononuclear cells were isolated from the spleen by Ficoll-Hypaque density gradient separation. B cells were enriched by panning, using plastic dishes coated with α-Thy-1 mAb ascites (NIM-R1). For activation, B lymphocytes were activated with *Escherichia coli* O55:B5 LPS (Sigma) at 40 µg/µl and 10 U/ml interleukin 4 (IL-4) (R&D Systems, Minneapolis, MN, USA) for 48 h at 37°C. For staining, the cells were washed with phosphate-buffered saline (PBS), fixed for 15 min with 4% paraformaldehyde (PFA), permeabilized with 0.1% Triton X-100, and finally incubated with different Abs or fluorescent reagents as previously described by Maravillas-Montero et al. (33).

### Induction of Capping

Primary enriched B cells were treated with NIM-R8, α-IgM (Zymed), α-transferrin receptor (TfR) (Santa Cruz Biotechnology) or HA (Sigma), and incubated for 15 min at 4°C. The cells were then washed with cold PBS and α-Rat-PE or α-Goat-PE, incubating for 60 min at 37°C to induce cross-linking with the antibodies. To stimulate crosslinking with HA, the cells were incubated for 30 min at 37°C with HA-FITC. Finally, the cells were fixed with 4% PFA and permeabilized with 0.1% of Triton X-100 to detect Myo1g, caveolin, or GM1 as described below. After staining, the cells were mounted on coverslips treated with poly-l-lysine (Sigma) for microscopy observations. In some experiments, cholesterol was removed in B lymphocytes. Briefly, cells were washed twice with 1× PBS and treated with 5 mM of MβCD (Sigma) for 15 min at 37°C (15, 17) or treated with MβCD plus cholesterol or lanosterol at 1 µg/ml for 30 min at 37°C. To block the recycling of adhesion molecules, the cells were washed twice with 1× PBS and treated with 50 µM of chloroquine (16), 0.3 mM of primaquine (39), or 50 µM of Golgi-stop (monensin) (40) for 1 h at 37°C.

### Immunoprecipitation

Resting, activated, or capping induced-B cells were lysed with RIPA buffer [20 mM Tris–HCl (pH 7.5), 150 mM NaCl, 1 mM EDTA, 1 mM EGTA, 1% Triton X-100, 1 µg/ml leupeptin, 10 µg/ml aprotinin, and 1 mM PMSF] 30 min at 4°C. After that, the lysates were centrifuged 30 min at 4°C at 18,000 *g* and the supernatants were mixed with α-Myo1g or NIM-R8, using rabbit IgG or rat IgG as isotype controls, respectively. The supernatants were incubated 4 h at 4°C in agitation; then, the complexes were precipitated with protein G-agarose (Life Technologies), maintaining the temperature at 4°C. Complexes were washed three times with RIPA buffer and boiled in Laemmli buffer. Western blotting was performed under standard conditions.

### Lipid Raft Isolation

For lipid raft isolation, 1 × 10^8^ B lymphocytes from WT or Myo1g^−/−^ mice (treated or not with MβCD) were washed with ice-cold PBS and lysed 30 min on ice with 1% Triton X-100 in TNE buffer containing protease and phosphatase inhibitors (TNE: 10 mM Tris/HCl, pH 7.5, 150 mM NaCl, and 5 mM EDTA plus 2.5 mg/ml each of PMSF, leupeptin, aprotinin, 50 mM sodium fluoride, and 1 mM sodium orthovanadate). The mixture was further homogenized with 10 strokes in a Wheaton loose-fitting dounce homogenizer. Nuclei and cellular debris were pelleted by 10 min centrifugation at 900 *g*. For the discontinuous sucrose gradient, 1 ml of cleared supernatant was mixed with 1 ml of 85% sucrose in TNE and transferred to the bottom of a Beckman 14 mm × 95 mm centrifuge tube. The diluted lysate was overlaid with 6 ml 35% sucrose in TNE and finally 3.5 ml 5% sucrose in TNE. The samples were centrifuged 20 h at 200,000 *g* and 4°C. Fractions of 1 ml were collected from the top of the gradient and then analyzed by WB.

### FACS Assays

Resting or activated B-lymphocytes from WT or Myo1g^−/−^ mice were harvested, then, these cells were washed and adjusted at 1 × 10^6^ cells/ml in ice cold 5% BSA in PBS and incubated 20 min on ice. After 5 min centrifugation at 400 *g* at 4°C, 1 µg/ml of specific primary antibody or subunit B of cholera toxin was added and incubated 20 min at 4°C in the dark. The cells were washed with PBA (PBS + 1% BSA + 0.1% NaN_3_) twice by centrifugation at 400 *g* for 5 min; then, the secondary antibody or reagent was added (depending of each staining) and incubated 20 min at 4°C in the dark. After incubation, the cells were washed three times with PBA (as described above) and finally resuspended in 1 ml of ice cold 1% PFA. The cells were analyzed by FACS or by confocal microscopy (depending of the experiment design).

### CD44 Recycling Assay

Mononuclear cells were isolated from the spleen by Ficoll-Hypaque density gradient separation, these cells were washed three times with cold PBS, and incubated for 30 min on ice with 1 mM of Lc-Biotin (Pierce, Rockford, IL, USA) in PBS. The reaction was terminated with the addition of 100 mM glycine (Sigma), incubating for 15 min on ice followed by extensive washing with cold PBS. Biotin-labeled cells were incubated with NIM-R8 followed by α-Rat-PE to induce CD44 internalization (as described above). Next, the cells were treated 30 min at 37°C with 0.25% trypsin (Gibco, Grand Island, NY, USA) to remove surface proteins, followed by the addition of 5 µg of unlabeled streptavidin (Sigma) and α-CD44 (clone IM7) to block the remaining biotinylated CD44 on the surface. Finally, the cells were incubated 10, 30, or 60 min at 37°C to allow the recycling of CD44. After completion of the treatment, the cells were fixed with PFA, stained with streptavidin-FITC (Sigma), α-CD44-PE (BioLegend), and α-B220-VB421 (BioLegend) and analyzed by flow cytometry.

### Proteins Associated to CD44 during Recycling

As described, B cells were labeled with biotin and then treated with α-NIM-R8 and α-Rat IgG to induce the internalization of CD44. The remaining biotinylated CD44 on the surface was removed with trypsin 0.25% and 5 µg of unlabeled streptavidin. The cells were extensively washed with PBS and then lysed with RIPA buffer. Biotinylated proteins were precipitated by streptavidin-agarose (Sigma) overnight at 4°C. After washing three times with RIPA buffer, the streptavidin-agarose beads were mixed with Laemmli buffer and the proteins were resolved in 12% SDS-PAGE gel and transferred to nitrocellulose membrane for immunoblot analysis using α-CD44 (Clone KM201).

### Spreading Assays

Glass slides were coated overnight at 37°C with NIMR8 in PBS. The coverslips were blocked with PBS containing 10% fetal calf serum for 1 h at 37°C and then, washed thoroughly with medium before use. B-lymphocytes from WT or Myo1g^−/−^ mice were transferred without washing to the precoated glass slides and incubated for different time intervals at 37°C. After incubation, the cells were fixed with 4% paraformaldehyde for 15 min, washed with PBS, and permeabilized with 0.1% Triton X-100 in PBS for 10 min. After permeabilization, the cells were washed with PBS, stained with TRITC-phalloidin, and mounted as described previously (41). The slides were observed in a FV300 Olympus microscope, using 60× objectives and the images were analyzed by NIH ImageJ software (http://rsbweb.nih.gov/ij).

### 2D Migration Assay

A Zigmond chamber (Neuro Probe, Inc., Gaithersburg, MD, USA) was used to evaluate migration. Briefly, one million B-lymphocytes were suspended in 0.5 ml of RPMI 1640 supplemented with 10% FBS (Life Technologies, Carlsbad, CA, USA) and immediately plated onto glass coverslips previously coated with 2.5 µg/ml of fibronectin (Takara). The coverslips were incubated 30 min at 37°C in 5% CO_2_ to allow cell attachment. The coverslips, with the cells attached, were gently washed with 2.0 ml PBS. One hundred microliters of supplemented RPMI 1640 was pipetted onto the coverslips, which in turn, were placed upside-down onto the Zigmond slides. One of the grooves, in the Zigmond chamber, was filled with supplemented medium (80 µl) and the chamber was placed under a microscope. A baseline image was obtained at 20× magnification and the other groove was then filled with CXCL13 (50 ng), also dissolved in supplemented medium. Digital images of the cells were taken every 20 s for 30 min, maintaining the temperature of the room at 35–39°C. Migration tracks were traced for at least 30 lymphocytes of each condition, in three independent experiments, using NIH ImageJ software with chemotaxis and migration tool 2.0 (Ibid). From these data, trajectories and migration speed were calculated.

### Hyaluronic-Acid Endocytosis Assay

B-cells were incubated 60 min at 4°C with HA–FITC, and then incubated at 37°C during different intervals of time before fixation. Levels of cell-associated HA–FITC were determined by FACS analysis, using a CyAn ADP flow cytometer (Beckman Coulter); the flow cytometry data were analyzed using FlowJo software.

### Nucleofection of B-Lymphocytes

The nucleofection of primary B lymphocytes was performed under manufacturer conditions (Lonza VPA-1010 kit). Briefly, panning-enriched B-lymphocytes were activated with LPS overnight. After that, the cells were placed at room temperature with B cell nucleofection solution (3 × 10^6^ B cells per 100 µl). Five micrograms of Full-Length Myo1g—Green Fluorescent Protein (Myo1g-FL) plasmid were added. The mixture was immediately transferred into an Amaxa cuvette and Nucleofector Amaxa Z-001 program was used. Nucleofected B-lymphocytes were placed in pre-warmed supplemented RPMI containing LPS + IL4, the cells were incubated 48 h at 37°C in 5% CO_2_.

### Endothelial Transmigration

Transwell chamber filters (5 µM, Corning) or 24-well plates (control) were coated with an attachment factor (Gibco) for 1 h at 37°C. The solution was then removed and the filters were dried. These filters were seeded with 2.5 × 10^4^ endothelial MLEC cells (42) per filter in 200 µl of complete DMEM. Two wells out of a 24-well plate were used to monitor the confluency of MLEC cells and the integrity of the endothelial barrier. The cells were incubated for 2 days at 37°C. When the cells reached confluency, the monolayers were treated with 5 nM TNF-α overnight. After incubation, the filters were washed with DMEM and the top and the bottom chambers were filled with 50 and 600 µl DMEM (without FBS), respectively. CXCL13 (1 µg/µl) was added in some of the bottom chambers. In some cases, CFSE-labeled resting or activated B cells were pretreated with α-CD44 clone IM7 for 1 h or left untreated. Then, 4 × 10^5^ B cells were added on top of each filter and incubated for 4 h. The filters were then carefully removed and the cells at the top and the bottom of each well were stained with α-B220-PB and analyzed by flow cytometry.

### *In vivo* Homing Assay

LPS plus IL-4 activated B cells from Myo1g^−/−^ or WT mice were labeled with either 0.1 µM (low concentration) or 0.6 µM (high concentration) CFSE (Invitrogen); in some experiments, these cells were preincubated with α–CD44 clone IM7. The cells were mixed at a 1:1 ratio and injected, *via* tail vein (at 1 × 10^7^ cells per mouse) into recipient mice (C57BL/6). Mice were sacrificed 2 h after the adoptive transfer, and spleens and inguinal lymph nodes were excised. B cells were surface stained with α-B220-PB (BioLegend). The cells were analyzed on a CyAn ADP flow cytometer (Beckman Coulter) for the presence of labeled cells and for expression of the B220 marker. The recovery ratios of each population were calculated as percentages using FlowJo software. To validate the procedure, in each experiment, Myo1g^−/−^ only or WT only cells were labeled with both high and low concentrations of CFSE and injected into recipient mice. Ratios close to 1:1 of the high and low CFSE labeled cells in the lymph nodes of the recipient mice indicate that the homing behavior of the cells was not significantly changed by the dye.

### Uptake Assays

Panning-enriched B cells (1 × 10^6^ cells per well in 500 µl of supplemented RPMI 1640 medium) were incubated for 30 min at 37°C in 5% CO_2_ in 24-well plates with HA coated 0.5 µm polystyrene fluorescent microspheres (43) (Molecular Probes), at bead-to-cell ratio of 10:1. The cells were then harvested and washed with PBS before the addition of 0.5 ml of a Trypan Blue solution (50 µg/ml) to quench the fluorescence of uningested particles. After a brief incubation for 2 min, the dye was extensively washed with PBS and cells were stained with α-B220-PB. The cells were then fixed and analyzed on a CyAn ADP flow cytometer (Beckman Coulter). In some experiments, the cells were treated with α–CD44 monoclonal antibody, clone IM7.

### Adhesion Assays

Hyaluronic acid (Sigma) or α-IgM (Zymed) coated 96 well polystyrene plates (Nalge Nunc International) were incubated for 1 h at 37°C. The plates were then washed twice with PBS before adding 4 × 10^5^ panning-enriched B cells in 200 µl of RPMI 1640 per well. The cells were adhered for 1 h at 37°C. The plates were then washed with 300 µl of PBS, fixed with 4% paraformaldehyde for 10 min, before adding crystal-violet (7.5 g/l crystal-violet, 2.5 g/l NaCl, 1.57% formaldehyde, 50% methanol) for an additional 5 min. The cells were washed extensively eight times with distilled water, solubilized with 10% SDS, and the amount of dye remaining in the plates was recorded at 540 nm (Multiskan Ascent, Thermo Scientific, Waltham, MA, USA). After subtraction of the non-specific dye bound to empty wells, absolute binding was calculated. The absorbance was determined in at least four wells per condition, in three independent experiments.

### Statistical Analysis

Results are presented as mean ± SD. An unpaired two-tailed Student’s *t*-test or one-way ANOVA were used to assess statistical significance between groups. The *P* values obtained and the number of samples or cells (n) used are mentioned in each figure legend.

## Results

### Myo1g Deficiency Affects the Polarization of CD44 in B Cells

To determine whether the absence of Myo1g affects the polarization of CD44 (capping), B cells from wild type (WT) and Myo1g^−/−^ mice were evaluated. We used LPS plus IL-4 activated-B lymphocytes in most experiments, because in non-activated B lymphocytes, it is difficult to record morphological changes induced by CD44 crosslinking or HA treatment (33, 37, 41, 44, 45). The activation triggers several signaling pathways; among others, STAT6 activation has shown to increase the number and length of membrane-protrusions, as well as the motility, of B lymphocytes (46).

**Figure 1:**
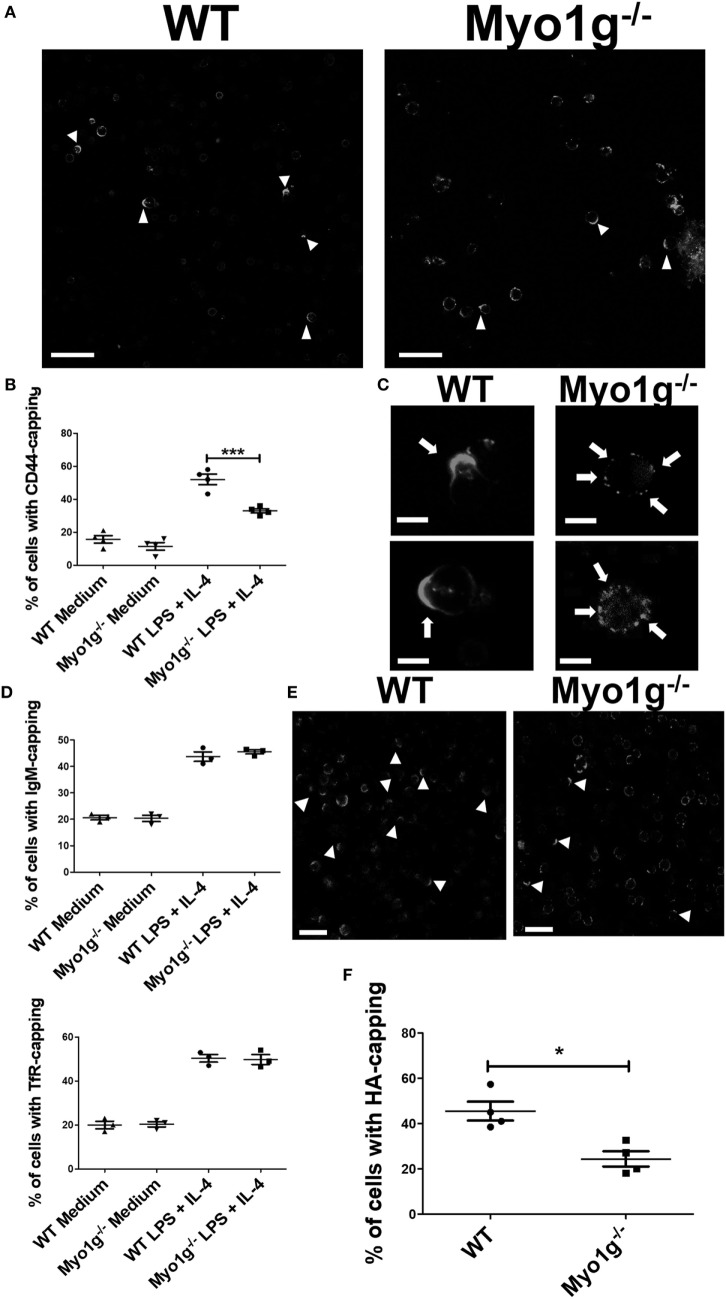
The absence of Myo1g affects the polarization of CD44 in B cells. **(A)** Confocal images of LPS plus IL4-activated WT or Myo1g^−/−^ B cells. Arrows illustrate CD44 localization in B lymphocytes (scale bar 60 µm). **(B)** Percentage of cells with CD44-capping (50 cells were counted per experiment in four independent experiments, 200 cells per data set). **(C)** Enlarged confocal images of LPS plus IL4-activated WT or Myo1g^−/−^ B cells. Arrows illustrate CD44 localization in B lymphocytes (scale bar 5 µm). Graphs of transferrin receptor (TfR), or IgM-capping (50 cells were counted per experiment in three independent experiments, 150 cells per data set). **(D)** Images of randomly selected files were taken and the percentage of B lymphocytes with capping was calculated (50 cells were counted per experiment in 4 independent experiments, 200 cells per data set). One-way ANOVA tests were used in **(B,D)** graphs, values are mean ± SD (***P < 0.001). **(E)** Confocal images of LPS plus IL4-activated from WT or Myo1g^−/−^ B cells. Arrows illustrate hyaluronic acid (HA) localization in B lymphocytes (scale bar 60 µm). **(F)** Percentage of cells with HA-capping; images of randomly selected files were taken, and the percentage of B lymphocytes with capping was calculated. A total of 200 cells was analyzed. Student’s *t*-test test was used in this graph, values are mean ± SD (50 cells were counted per experiment, in four independent experiments, 200 cells per data set) (*P < 0.05).

The cells were stimulated 48 h with LPS + IL-4 and then incubated with the NIM-R8 antibody to induce CD44 polarization. Confocal microscopy demonstrated that CD44 had reduced mobility to the site of crosslinking in cells lacking Myo1g (Figure 1A, white arrows). To quantify these defects, the percentage of cells showing polarized structures was determined, demonstrating a reduction in the percentage of cells with full caps in Myo1g-deficient B cells (Figure 1B). In fact, the distribution of CD44 in Myo1g-deficient B cells was scattered in patches (Figure 1C, white arrows). The same experiments were performed for several molecules such as TfR (47) and IgM (48–53). The results demonstrated that these molecules did not show defects in their polarization (Figure 1D). CD44 is the main receptor of HA and this extracellular matrix component induced a strong capping in lymphocytes (54); hence, HA-capping assays were performed for both groups of B lymphocytes. A similar pattern of scattered patches as in Myo1g-deficient B lymphocytes was also observed (Figure 1E). To quantify these defects, the percentage of cells showing capping was determined, demonstrating a reduced percentage of cells with full caps (Figure 1F).

It was noted that the size of the cap in Myo1g-deficient B cells was larger than that in WT B lymphocytes (Figure S1A in Supplementary Material). Therefore, the size of the cap was calculated through an index in which the extent of the cap was divided between the circumference of the cell. This parameter indicated the agglomeration of molecules at that site. The results showed that during capping, Myo1g-deficient B cells have a less uniform distribution of CD44 than WT B cells (Figure S1B in Supplementary Material).

Several studies have demonstrated the importance of the actin-cytoskeleton in the polarity of molecules (55–57). Therefore, we evaluated the polarization of actin to the CD44 capping site. The results demonstrated the accumulation of actin to the CD44 capping site (Figure S2A in Supplementary Material). Cytochalasin E (a drug that blocks polymerization and depolymerization at the barbed ends of actin filaments) was also used to examine the role of actin polarization in CD44 capping. Cytochalasin E treatment negatively affected CD44-polarization (Figure S2B in Supplementary Material). This effect was quantified showing an almost complete inhibition of CD44 capping by Cytochalasin E (Figure S2C in Supplementary Material).

**Figure 2:**
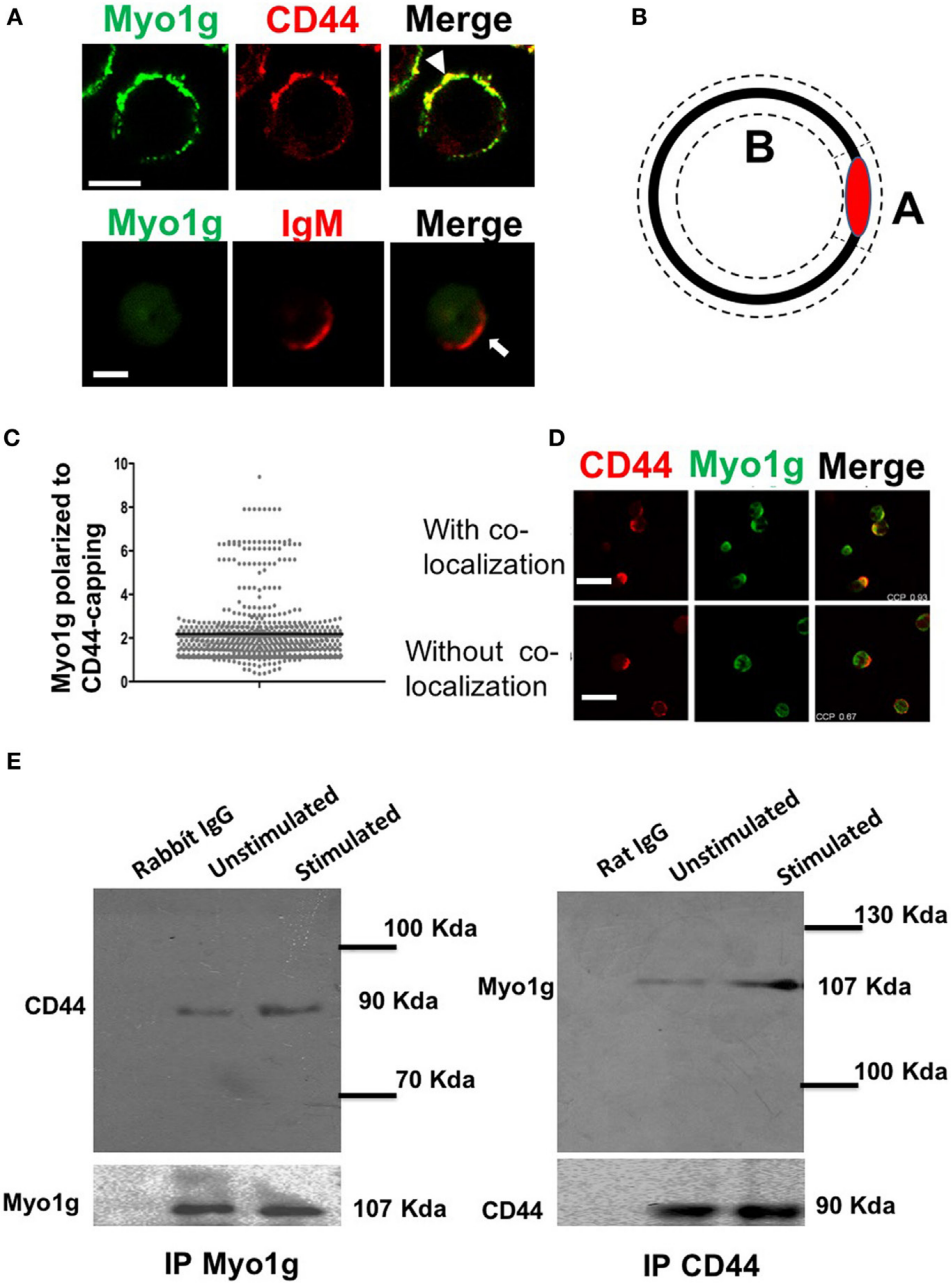
Myo1g is polarized to the capping site of CD44. **(A)** Confocal images of LPS plus IL4-activated WT or Myo1g^−/−^ B cells. Arrows indicate polarization of Myo1g at the CD44-capping site (scale bar 5 µm). **(B)** Schematic illustration to calculate the polarization index, where A corresponds to the mean fluorescence intensity (MFI) in the polarized region, and B is the MFI in the rest of the cell. The MFI from A is divided by B. If the value is greater than 1.5, the co-localization is considered positive; values less than 1.5 are considered negative. **(C)** Polarization coefficient of Myo1g and CD44 derived from the analysis of B lymphocytes (~80 cells were counted per experiment, in 3 independent experiments, 250 cells per data set), values are mean ± SD. **(D)** Confocal images of B cells with CD44-capping (scale bar 60 µm). **(E)** Immunoprecipitation with α-Myo1g or α-CD44.

Previous studies have demonstrated the accumulation of myosins at the gathering sites of several molecules (41). To investigate whether Myo1g actively participates in the mobilization of CD44, cross-linking with the NIM-R8 antibody was performed, to find Myo1g at the site of polarization. Most cells showed Myo1g at the CD44 cap. In contrast, Myo1g did not congregate with IgM (Figure 2A). To evaluate polarization, an index of the mean fluorescence intensity (MFI) in the cap divided by the MFI outside the cap was calculated—this coefficient represents the amount of Myo1g polarized to CD44-capping site (Figure 2B). Indexes above 1.5 represented positive co-capping. With the crosslinking of CD44, many cells showed positive co-capping (Figure 2C). In some cells, Myo1g did not co-localize on the cap but rather on the opposite side of the cell (Figure 2D). The coefficient of co-localization between CD44 and Myo1g showed that 63% of lymphocytes presented co-clustering of both molecules (data no shown). This indicates a selective role of Myo1g in the mobilization of CD44, which contrasts with IgM polarization. This mobilization appears to be independent of lipid raft clustering (56).

Then, to evaluate if Myo1g was present in the mobilization complexes of CD44, immunoprecipitation assays for Myo1g and CD44, in resting and activated lymphocytes, were performed. The results demonstrated that Myo1g co-immunoprecipitated with CD44 and *vice versa* (Figure 2E). To further analyze this association, activated B lymphocytes were stimulated with HA (its natural ligand) and then, CD44 was immunoprecipitated to search if Myo1g was increased when CD44 was bound with its ligand. As shown Figure S3A in Supplementary Material, Myo1g is associated with CD44 irrespective of ligand occupancy.

Studies indicate that recycling is required for the polarization of some surface molecules (58–61). We analyzed the internalization of CD44 using HA as ligand, we observed the reduction of CD44 on the plasma membrane at 10 min; however, the cells partially recovered CD44 levels from 20 min. These data suggest a recycling of CD44 in response to HA binding (Figure S3B in Supplementary Material). Next, B lymphocytes treated with primaquine, monensin (Golgi-stop), or chloroquine [drugs that blocks cellular recycling (39, 62–67)] were analyzed for CD44-capping. The results showed impaired formation of CD44-caps in drugs-treated cells (Figure S4A in Supplementary Material). To quantify the effect of reagents, the percentage of cells showing capping was determined, demonstrating a significant reduction (Figure S4B in Supplementary Material).

**Figure 3:**
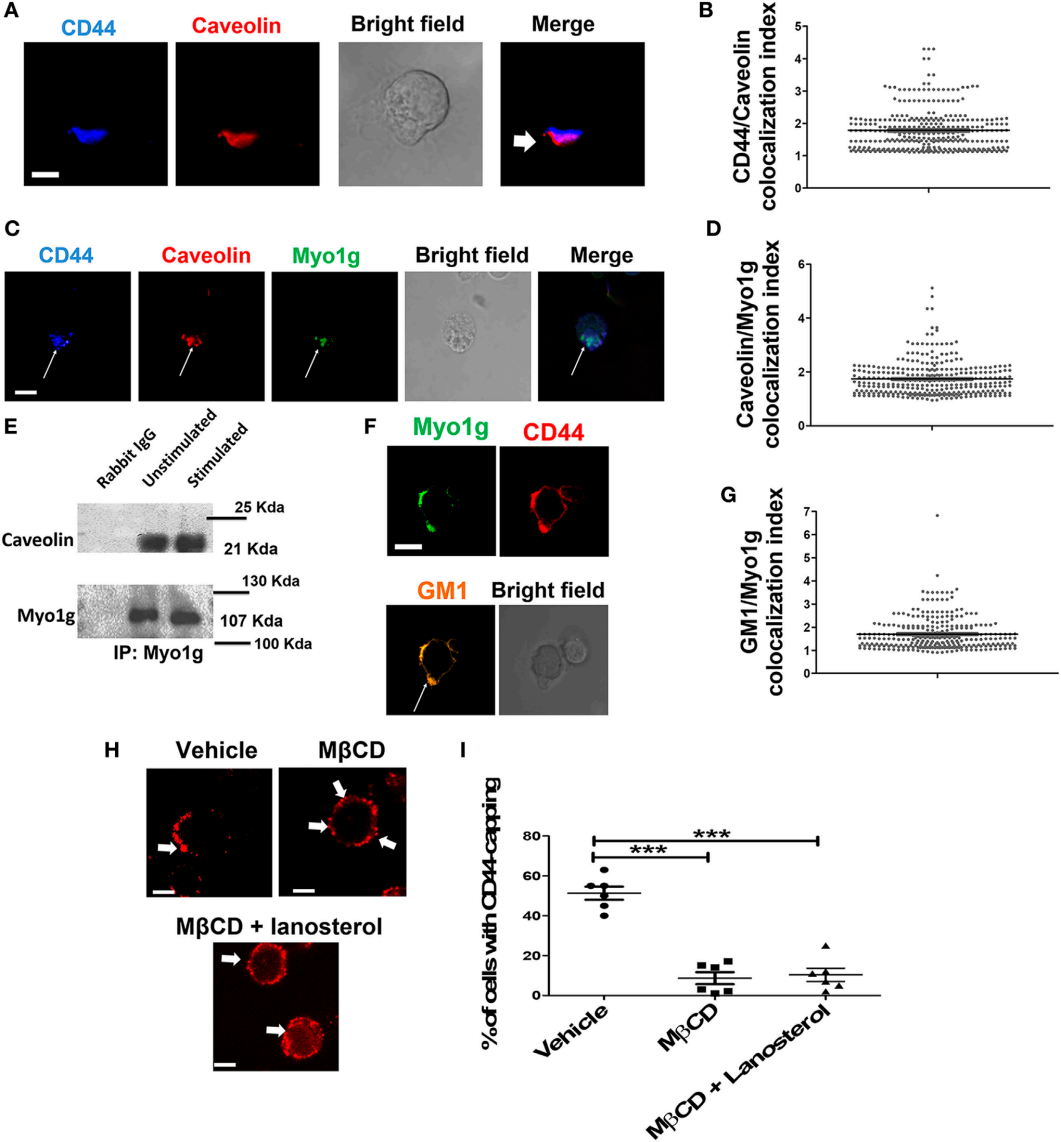
Myo1g is present in the mobilization complex of CD44. **(A)** Confocal images of LPS plus IL4-activated WT B cells. Arrows illustrate the polarization of caveolin to the CD44-interaction site (scale bar 5 µm). **(B)** Polarization coefficient of caveolin and CD44, derived from the analysis of a total of 300 cells with capping from three independent experiments (100 cells were counted per experiment), values are mean ± SD. **(C)** Confocal images of LPS plus IL4-activated WT B cells. Arrows illustrate the polarization of Myo1g and caveolin to the CD44-interaction site (scale bar 5 µm). **(D)** Polarization coefficient of caveolin and Myo1g (~80 cells were counted per experiment, in 3 independent experiments, 250 cells per data set), values are mean ± SD. **(E)** Immunoprecipitation with α-Myo1g or α-Rabbit (as isotype control) of LPS plus IL4-activated B cells. **(F)** Confocal images of LPS plus IL4-activated WT B cells. Arrows illustrate the polarization of GM1 (lipid rafts) to the CD44-interaction site (scale bar 5 µm). **(G)** Polarization coefficient of GM1 and Myo1g derived from the analysis of B lymphocytes (~70 cells were counted per experiment, in 3 independent experiments, 200 cells per data set), values are mean ± SD (*n* = 3). **(H)** Confocal images of LPS plus IL4-activated WT or Myo1g^−/−^ B cells. Arrows indicate the localization of CD44 in B cells pretreated with MβCD (5 mM) with or without lanosterol (1 µg/µl) (scale bar 5 µm). **(I)** Percentage of cells with CD44-capping. Images of randomly selected files were taken and the percentage of B cells with capping was evaluated (*n* = 6), one-way ANOVA test was used in this graph values are mean ± SD (***P < 0.001).

Previous reports have shown that mobilization of CD44 during cell migration is caveolin- and lipid rafts-dependent. Therefore, we analyzed caveolin and CD44 co-capping in B cells. The results showed the presence of caveolin at the gathering site of CD44 (Figure 3A). Based on the polarization index described above, an increase in caveolin MFI was observed in the CD44-cap (Figure 3B). Furthermore, Myo1g co-localized with caveolin (Figure 3C). We also calculated the Myo1g-caveolin polarization index, which was above 1.5 in most cells (Figure 3D). To further corroborate these results, an immunoprecipitation assay of Myo1g, using α-CD44-crosslinked B cells was performed. The presence of caveolin in the immunoprecipitated fraction was observed, indicating the presence of CD44, caveolin, and Myo1g in the same fractions (Figure 3E).

During capping, lipid rafts are important in the mobilization of several molecules (68–71). Some studies have demonstrated a strong interaction between caveolin and lipid rafts (72–76). As a surrogate for lipid rafts, GM1 was identified using subunit B of cholera toxin (CTB) during CD44-capping. GM1 was associated with CD44 and Myo1g (Figure 3F). The Myo1g-GM1 polarization index was calculated, showing a polarization index above 1.5 in most cells (Figure 3G). To complement and extend these results, CD44-capping was induced with HA, the natural ligand of CD44, and Myo1g, caveolin, and lipid rafts (GM1) were searched at the HA-capping site (white arrows, Figure S5B in Supplementary Material). Colocalization index was quantified in Figure S5B in Supplementary Material.

To further evaluate the role of lipid rafts in the mobilization of CD44, B cells were treated with methyl-β-cyclodextrin (MβCD, cholesterol-sequestering drug). Cholesterol-depleted cells were stimulated with the NIM-R8 antibody plus α-Rat IgG-PE and visualized by confocal microscopy. Cholesterol-depleted cells showed less polarization of CD44. Notably, most cells did not present CD44-capping (Figures 3H,I). Additionally, we treated cholesterol-depleted B lymphocytes with lanosterol [a sterol that replaces cholesterol in plasma membrane but does not support the lipid rafts formation (77)]. Replacement with lanosterol showed an impaired formation of capped-structure (Figures 3H,I). Interestingly, the pattern of CD44 localization was in patches (white arrows), a strikingly similar phenotype to that observed in Myo1g-deficient B cells (Figure 1C). To corroborate the crucial role of lipid rafts in CD44-polarization in B-lymphocytes, we added cholesterol to MβCD-treated cells, using 5 mM MβCD and 1, 2, and 5 µg/µl concentrations of cholesterol. As depicted in Figure S6 in Supplementary Material, CD44-polarization was recovered when cholesterol was added. Of note, cholesterol at higher concentrations seems to partially interfere with CD44-polarization. It is known that the increase in cholesterol drives alterations in plasma membrane rigidity (78), this modification may induce changes in shape, adhesion, locomotion (79), and probably vesicular traffic of adhesion molecules (such as CD44) in the cells. We analyzed spreading of B cells (another process in which lipid rafts are crucial), using MβCD-treated B lymphocytes reconstituted or not with cholesterol or lanosterol. We registered reduction of spreading in MβCD-treated B cells; in contrast, MβCD-treated B cells, reconstituted with cholesterol but not with lanosterol recovered the ability to spread and to form dendrite. Cholesterol, on its own, reduced spreading of B-cells (Figures S7A,B in Supplementary Material).

### The Absence of Myo1g Redistributes CD44 from the Cell Surface to Intracellular Membranes

**Figure 4:**
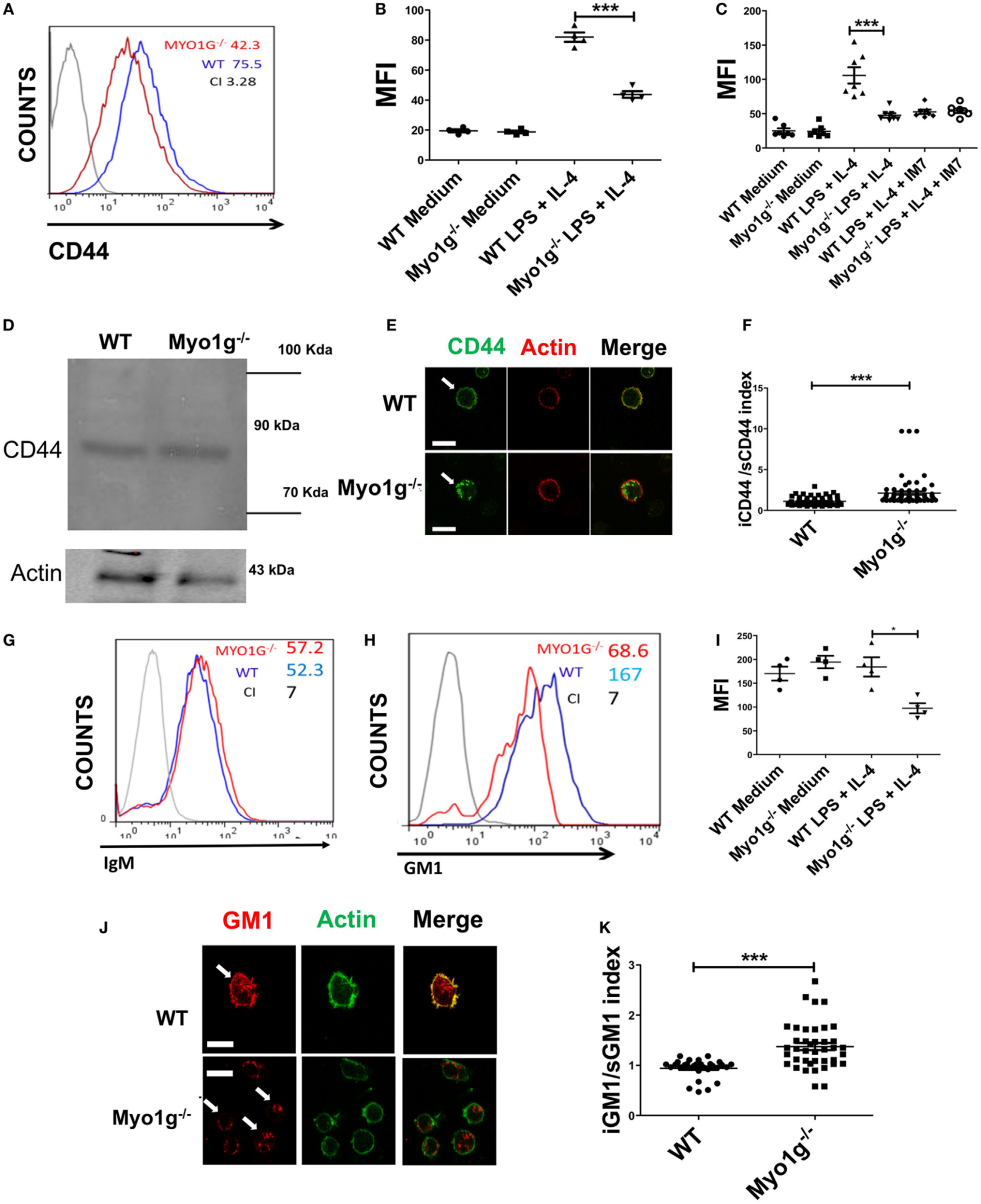
The absence of Myo1g decreases the amount of CD44 and lipid rafts on the surface of B cells. **(A)** Expression of CD44 in LPS plus IL4-activated B cells from WT or Myo1g-deficient mice. The graph shows the expression of CD44 in B lymphocytes (10,000 events in a gate of B220 + B cells). **(B)** Expression of CD44 in resting or activated B cells, one-way ANOVA test was used in these experiments, values are mean ± SD (***P < 0.001) (*n* = 4). **(C)** Binding of hyaluronic acid (HA-FITC) to resting or LPS plus IL4-activated B lymphocytes from WT or Myo1g-deficient B lymphocytes. HA binding was blocked using the IM7 mAb. One-way ANOVA test was used in these experiments, values are mean ± SD (***P < 0.001) (*n* = 7). **(D)** Immunodetection of CD44 in LPS plus IL4-activated B lymphocytes; α-CD44 (clone KM201) was used for western blotting. **(E)** Confocal images of LPS plus IL4-activated WT or Myo1g^−/−^ B cells. **(F)** The localization of CD44 as cytosol/plasma membrane index was calculated [mean fluorescence intensity (MFI) in the plasma membrane was divided by MFI in the cytosol] (~30 cells were counted per experiment, in three independent experiments, 100 cells per data set). Student’s *t*-test was used in these experiments, values are mean ± SD (***P < 0.001). **(G)** IgM or **(H)** GM1 in LPS plus IL4-activated B lymphocytes from WT or Myo1g-deficient B cells, the graphs show the expression of GM1 in B lymphocytes (10,000 events in a gate of B220 + B cells). **(I)** GM1 staining, one-way ANOVA test was used in these experiments, values are mean ± SD (*P < 0.05) (~60 cells were counted per experiment, in four independent experiments, 250 cells per data set). **(J)** Confocal images of permeabilized LPS plus IL4-activated WT or Myo1g^−/−^ B cells. Arrows indicate the localization of GM1 in B lymphocytes (scale bar 5 µm). **(K)** Intracellular versus plasma membrane GM1 (~30 cells were counted per experiment, in 3 independent experiments, 100 cells per data set), Student’s *t*-test was used in these experiments, values are mean ± SD (***P < 0.001).

In a previous study, we showed that Myo1g is present in specialized lipid domains at the plasma membrane (33). During cell migration, CD44 is associated with microdomains. Therefore, we investigated the role of Myo1g in regulating CD44 trafficking and distribution. First, the localization of CD44 on the surface of Myo1g-deficient cells was evaluated. Resting B cells of both WT and Myo1g^−/−^ mice have similar amounts of CD44 on the surface. In sharp contrast, LPS plus IL4-activated B cells from Myo1g^−/−^ demonstrated a substantial decrease in CD44 on the plasma membrane, and this protein accumulated inside the cells (Figures 4A,B). Similar results were observed using HA-FITC to measure the levels of CD44 on the surface. B cells were pretreated with α-CD44 clone IM7, which blocks the interaction between CD44 and HA. Consequently, impaired binding of HA-FITC to B cells from Myo1g^−/−^ mice was observed (Figure 4C). Remarkably, the expression of CD44 in whole cells was similar (Figure 4D) indicating that the missing CD44 on the surface was retained inside the cell. This hypothesis was corroborated by confocal microscopy observations in permeabilized B lymphocytes (Figure 4E). To quantify the presence of CD44 inside or outside the cell, an intracellular/membrane index was calculated (Figure 4F). This index was quantified by MFI of CD44 inside, this value was divided between MFI of CD44 in surface. The data indicated an increased presence of CD44 inside Myo1g-deficient B cells. To rule out a generalized deficiency of membrane proteins, the presence of IgM on the cell-surface of both groups of B lymphocytes was analyzed, and both demonstrated similar expression of membrane proteins (Figure 4G).

Because the absence of functional Myo1g leads to a reduction in CD44 on the cell surface, and since this molecule is recycled in a lipid raft-dependent mechanism, the level of lipid rafts at the plasma membrane of Myo1g-deficient mice was analyzed. Activated and resting B lymphocytes were labeled with biotin-CTB and streptavidin (Figures 4H,I). Permeabilized B cells from Myo1g^−/−^ mice showed reduced staining with CTB compared to B cells from WT mice. Staining with CTB was observed inside Myo1g-deficient B lymphocytes (Figure 4J). The intracellular/plasma membrane index demonstrated an increased presence of intracellular GM1 in Myo1g-deficient B cells (Figure 4K). These data indicate that Myo1g deficiency affects the localization of lipid rafts. Taken together, these findings suggest a role for Myo1g in either recycling or exocytosis of lipid raft-enriched membranes from the intracellular membrane compartments back to the cell surface.

### Myo1g Participates in the Recycling Complex of CD44

**Figure 5:**
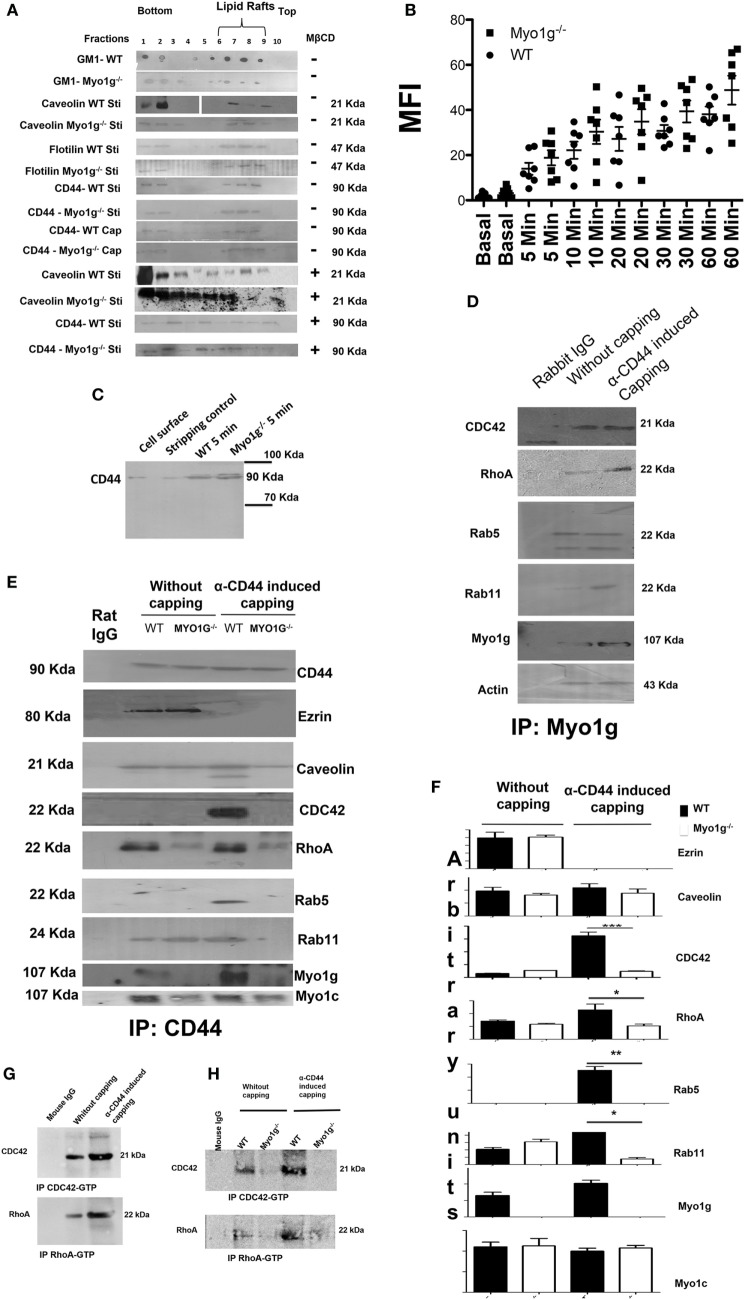
The absence of Myo1g alters the recycling of CD44. **(A)** Western blot of CD44, caveolin, flotilin, GM1 in activated or cap-induced B cells from WT or Myo1g-deficient B cells, treated or not with MβCD (*n* = 3). **(B)** Resting B- lymphocytes were treated with FITC-hyaluronic acid (HA) for 1 h at 4°C and were then incubated at 37°C for different time points. After incubation, the fluorescence was quenched by the addition of 0.5% trypan blue for 30 s, and the cells were extensively washed with 1× phosphate-buffered saline. Finally, the cells were fixed and fluorescence was analyzed by flow cytometry. The graph shows the percentage of HA endocytosed by B-lymphocytes. The results are derived from the analysis of seven independent experiments and presented as mean ± SD. **(C)** Immunodetection of intracellular CD44 in WT or Myo1g^−/−^ activated B cells induced to cap with the NIMR8 mAb (*n* = 3). **(D)** Immunoprecipitation of Myo1g in activated B lymphocytes from WT mice, comparing cells with or without CD44-induced capping (*n* = 3). **(E)** Immunoprecipitation of CD44 from activated B cells from WT or Myo1g-deficient mice, comparing cells with or without CD44 capping (*n* = 3). **(F)** Densitometry analysis of GTPases associated with the CD44-recycling complex. Images were analyzed using the ImageJ software to obtain the values. One-way ANOVA test was used in this graph, values are mean ± SD (*n* = 3) (*P < 0.05) (**P < 0.01). **(G)** Immunoprecipitation of CDC42-GTP and RhoA-GTP in activated or α-CD44-induced-capping B lymphocytes from WT mice (*n* = 3). **(H)** Immunoprecipitation of CDC42-GTP and RhoA-GTP from activated B cells from WT or Myo1g-deficient mice, comparing cells with or without CD44 capping (*n* = 3).

The reduction in the amount of CD44 on the membrane of B cells from Myo1g-deficient mice can be explained by defects in its recycling process. To test this, we evaluated different steps of the recycling process. The recruitment of CD44 to lipid rafts did not vary between WT and Myo1g-deficient B lymphocytes (Figure 5A), we can observe the lipid rafts region in 6, 7, and 8 fractions. An impaired amount of lipid rafts in Myo1g-deficient B lymphocytes was observed; this reduction can be explained by the reduced quantity of lipid rafts in the plasma membrane of activated B cells (Figure 4). We also treated B-lymphocytes with MβCD to destroy lipid rafts and then searched for CD44. We did not observe significant differences in the localization of CD44 between WT and Myo1g-deficient B lymphocytes (Figure 5A, bottom). We next evaluated if HA-endocytosis by B lymphocytes from WT or Myo1g-deficient was comparable. We detected similar internalization of HA in both groups (Figure 5B).

Continuing in this same way, we evaluate CD44-endocytosis in response to crosslinking, the following assay was developed. Surface proteins from B cells were biotinylated, and then 5-min capping with α-CD44 was induced. The cells were trypsinized, and then treated with unlabeled streptavidin to quench extracellular proteins and recover only the intracellular label. The cells were then lysed and the biotinylated proteins were immunoprecipitated using streptavidin-agarose. Finally, the presence of biotinylated-CD44 was detected in the intracellular compartments. Under these conditions, we detected only internalized protein, because we “shave” the cell surface, furthermore, we treated B lymphocytes with unlabeled streptavidin; in these circumstances, all the cell surface proteins were “unlabeled,” only biotinylated proteins inside the cell (because of the internalization) were “protected” and could be detected.

We observed similar endocytosis of CD44 in B cells from both WT and Myo1g-deficient mice in response to CD44-crosslinking (Figure 5C). The same experiments were repeated after 10-min capping (Figure S8A in Supplementary Material); or at lower temperature (Figure S8B in Supplementary Material); or with colchicine treated B-lymphocytes (Figure S8C in Supplementary Material) (80–83). We did not observe differences in CD44-internalization between WT and Myo1g^−/−^ B cells.

One important step in the recycling process is the association of GTPases (CDC42, RhoA, Rab5, and Rab11) with the recycling complex. During the recycling, GTPases change from an inactive to an active conformation (84). Accordingly, we evaluated the association of Myo1g with GTPases. The results demonstrated the presence of several GTPases in the immunoprecipitates of Myo1g (Figure 5D).

CD44 is involved in several processes such as adhesion, migration, and invasion. To perform these functions, CD44 must be internalized and recycled to the plasma membrane. This pathway involves GTPases such as Rab5, Rab4, Rab11, RhoA, and CDC42, as well as the coating protein caveolin (84) in several steps of this recycling pathway.

To analyze the participation of Myo1g in the recycling of CD44, immunoprecipitation assays using WT and Myo1g-deficient B cells were performed. A first step involves the migration of CD44 to lipid rafts through association with caveolin, dissociation from ezrin, and recruitment of RhoA and CDC42 to lipid rafts. A second step is defined by the association of the endosome carrying CD44 with Rab5 (early endosome). The third step is the association of this vesicle with Rab11. We demonstrate that the migration of CD44 to lipid rafts was similar between WT and Myo1g-deficient B cells. In addition, recruitment of RhoA and CDC42 was reduced in Myo1g-deficient B cells compared to WT cells. In agreement with this observation, the association of vesicles carrying CD44 with Rab5 was incomplete and there was no association with Rab11 (Figure 5E).

Myo1c has been implicated in this recycling pathway (85); therefore, we tested for the presence of this motor protein in this complex, in the absence of Myo1g. No differences in Myo1c association were observed. To quantify the differences in the molecules associated with the CD44-recycling complex, a densitometry analysis was performed (Figure 5F). The activation of CDC42 and RhoA in response to CD44-crosslinking was also evaluated. We immunopreciptated RhoA and CDC42 under the conditions mentioned in Figures 5D,E, finding an increase in the activation of these GTPases (Figure 5G). In sharp contrast, we found an impaired activation of RhoA and CDC42 in Myo1g-deficient B cells after CD44-crosslinking (Figure 5H).

**Figure 6:**
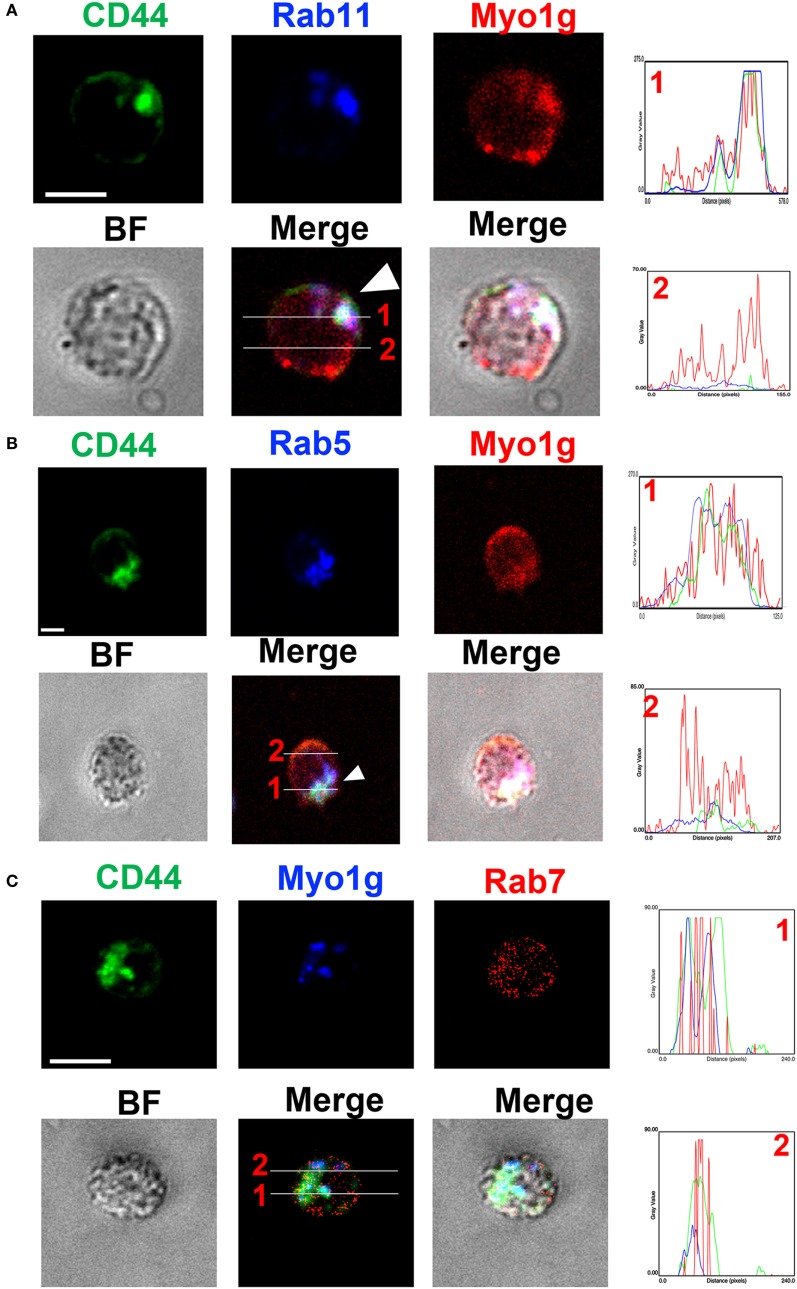

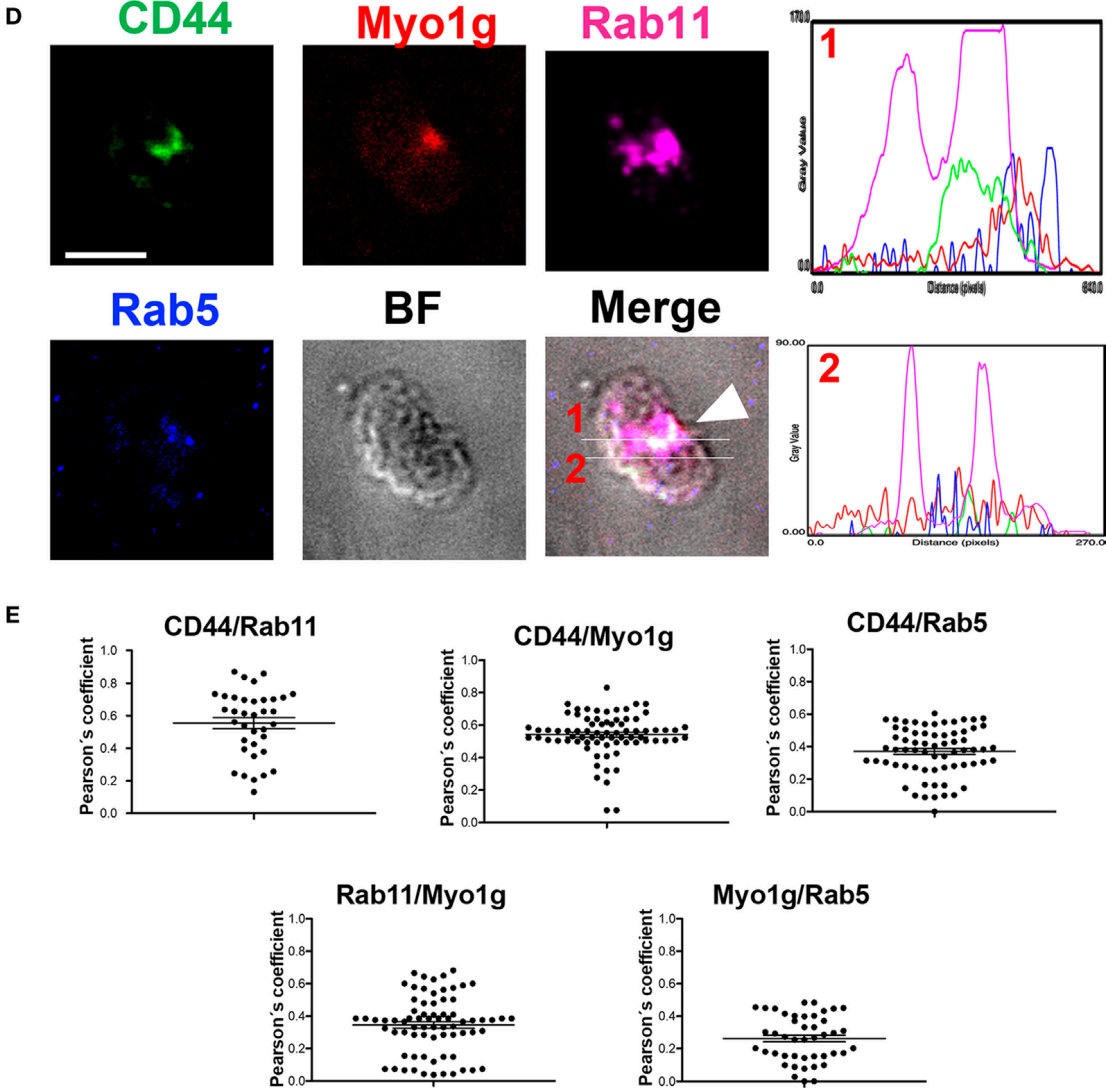
Myo1g interacted with Rab5 and Rab11. Confocal images of LPS plus IL4-activated WT B cells and then, crosslinked with α-CD44. The cells were stained with α-CD44, α-Rab5, α-Rab7, α-Rab11, and α-Myo1g. Arrows illustrate the location of the molecules during CD44-recycling phenomena (scale bar 5 µm), **(A)** Rab11, **(B)** Rab5, **(C)** Rab7, **(D)** Rab5 and Rab11. **(E)** Pearson’s correlation coefficient between several molecules.

In this same context, we performed staining for CD44, Myo1g, Rab5, Rab7, and Rab11 to validate the results mentioned above. We analyzed the cells 20 min after the NIMR8-crosslinking, we observed strong colocalization between Rab11, CD44, and Myo1g (Figure 6A); a more discreet interaction was observed with Rab5 (Figure 6B); however, we did not detect colocalization with Rab7 (Figure 6C). To further validate these data, the location of Rab5, Rab11, CD44, and Myo1g was evaluated, where these four cell molecules can be observed in the same pixels (Figure 6D). The profiles are shown in histograms next to figures, the lines indicates the location of cell molecules. Finally, the Pearson’s coefficient (colocalization index) between these molecules is depicted in Figure 6E. These values allow us to infer a spatiotemporal organization of CD44-recycling.

**Figure 7:**
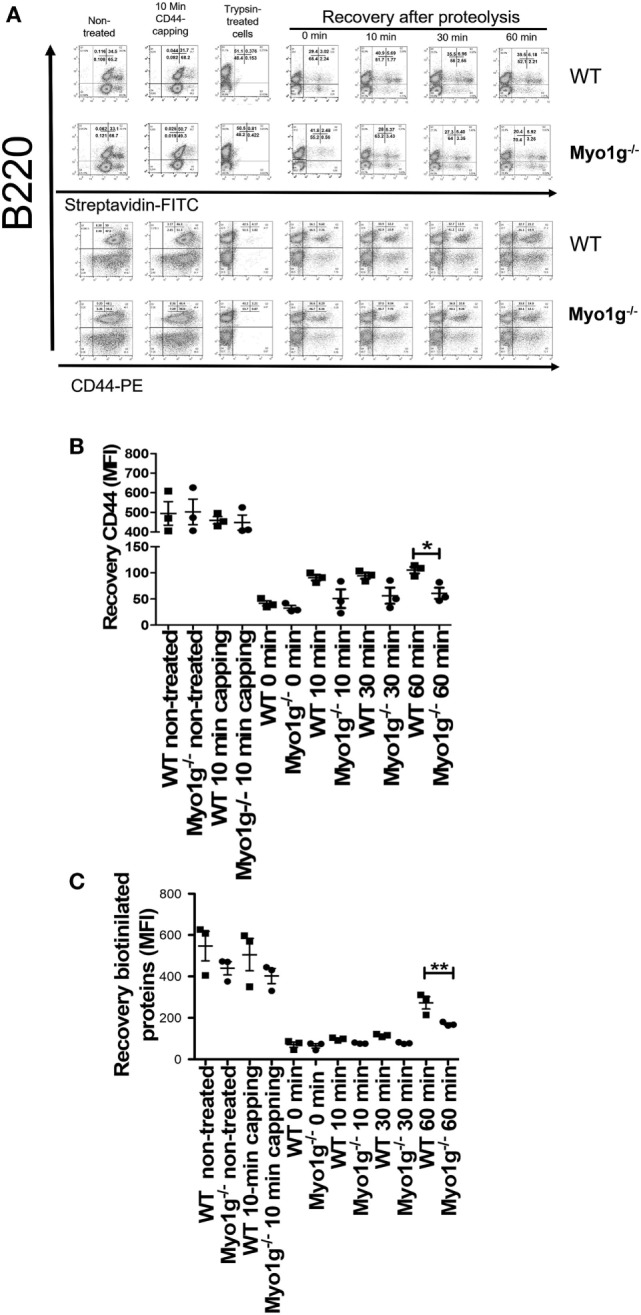
The absence of Myo1g delays the recycling of CD44. **(A)** Kinetics of CD44-recycling in B lymphocytes from WT or Myo1g-deficient mice. MFI of CD44 **(B)** or biotinylated proteins **(C)** in B lymphocytes during the recycling process, One-way ANOVA tests were used in these experiments, values are mean ± SD (*P < 0.05, **P < 0.01) (*n* = 3).

The results indicate that Myo1g plays a role in the output of lipid raft-associated proteins to the plasma membrane. To further confirm these observations, splenic lymphocytes were biotinylated, and then the cells were incubated 10 min with NIM-R8 antibody to induce the capping of CD44. After capping, these cells were treated with trypsin to remove surface proteins (survival rate after trypsin treatment was always 90–95%—data not shown). These trypsin-treated cells were further incubated to measure the rate of lipid raft recycling in B lymphocytes (using α-B220 antibody as generic marker to identify B cells). As shown in Figure 7A, the rate of endocytosis of these molecules was similar in both cell types; however, the exocytosis of CD44 (Figure 7B) and biotinylated proteins (Figure 7C) (in pre-gated B220^+^CD44^+^ and B220^+^Streptavidin^+^, respectively), was significantly lower in Myo1g-deficient B cells compared to WT lymphocytes. This reduced velocity of raft recycling caused a dramatic accumulation of labeled protein inside the cell.

**Figure 8:**
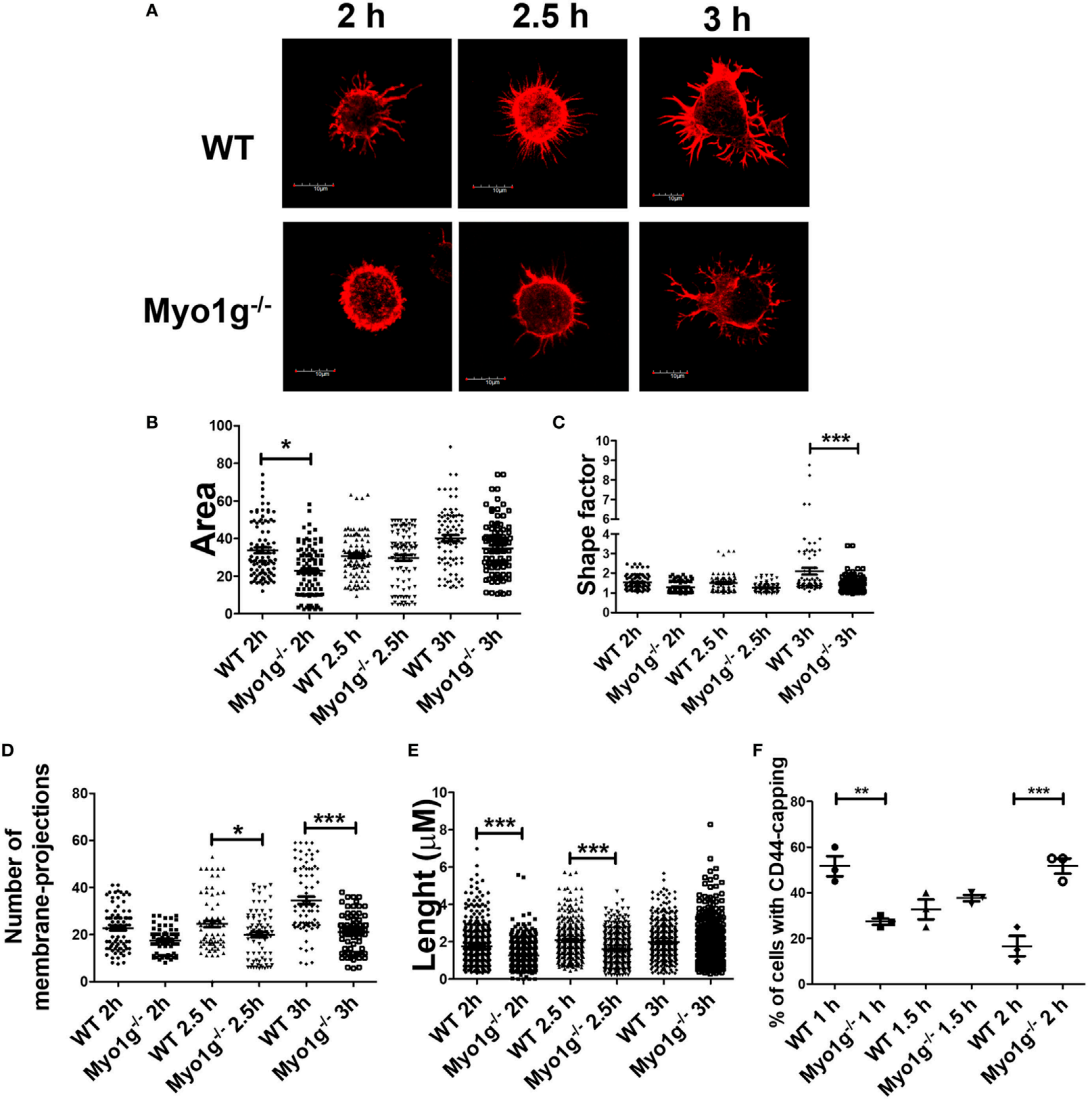
The absence of Myo1g delays the formation of membrane-protrusions in B lymphocytes. **(A)** Confocal images of activated B cells from WT or Myo1g-deficient mice. B lymphocytes with spread morphology were evaluated for **(B)** area, **(C)** shape, **(D)** number of projections, and **(E)** the length of these projections after 2, 2.5, and 3 h. A total of 100 cells, in three independent experiments, were analyzed in each group. **(F)** Percentage of cells with CD44-capping after 1, 1.5, and 2 h. Images of randomly selected files were taken and the percentage of B lymphocytes with capping was recorded (~70 cells were analyzed in 3 independent experiments, a total of 200 cells per data set are presented). One-way ANOVA tests were used in these experiments, values are mean ± SD (*P < 0.05, **P < 0.01, ***P < 0.001).

During spreading, recycling of CD44 is needed (86). To analyze how CD44 is relocated on the cell surface and how this affect spreading, the trypsin-treated cells, described above, were incubated for 2, 2.5, and 3 h and analyzed by their capacity to spread over CD44-coated coverslips. Cells in spreading have larger membrane-protrusions and elongated cell-morphology, contrasting with B-cells that did not spread, who have rounded morphology without visible protrusions (37). Although less Myo1g-deficient B cells adhered to the coverslips, those that bound to the surface showed a reduced number of membrane-extensions (observed though phalloidin-TRITC staining) at 2 and 2.5 h. However, after 3 h, B cells belonging to Myo1g-deficient mice produced membrane-protrusions like B cells from WT mice (Figure 8A). In these experiments, the area of the cells (Figure 8B), the shape factor (Figure 8C) as well as the number (Figure 8D), length (Figure 8E), and thickness (Figure S9A in Supplementary Material) of these membrane-projections were evaluated. Shape factor is a coefficient of the length and width of each given cell. Values closer to 1.0 indicated a more rounded morphology (33, 41, 44, 87).

The results showed a decrease in these parameters at 2 and 2.5 h. However, we detected a trend toward recovery, like the WT phenotype, within 3 h of adhesion. Another important characteristic of these B cells was the presence of branched protrusions in WT B cells. Myo1g-deficient B cells showed reduced percentage, numbers of ramifications by protrusion, and number of branched protrusions by cell (Figures S9B–D in Supplementary Material).

Like the spreading assay, when the incubation time for the capping assay was extended from 0.5 to 2 h, a recovery in the polarization of CD44 in Myo1g^−/−^ B lymphocytes was observed (Figures S9E,F in Supplementary Material). Finally, CD44 polarization in B lymphocytes from Myo1g^−/−^ mice without trypsin-treatment (see above) and extended incubation (1, 1.5, and 2 h) also showed delayed polarization of CD44 (Figure 8F).

To confirm the contribution of Myo1g in CD44-recycling, Myo1g^−/−^ B cells were transfected with Full-Length Myo1g coupled to GFP (Myo1g-FL) or with an empty vector. We examined the percentage of Myo1g^−/−^ expressing Myo1g-FL (Figure S10A in Supplementary Material). Around 55–70% of cells were positive to GFP. We also verified the expression of Myo1g *via* western blot, we observed the presence of Myo1g in Myo1g^−/−^ B cells (Figure S10B in Supplementary Material).

**Figure 9:**
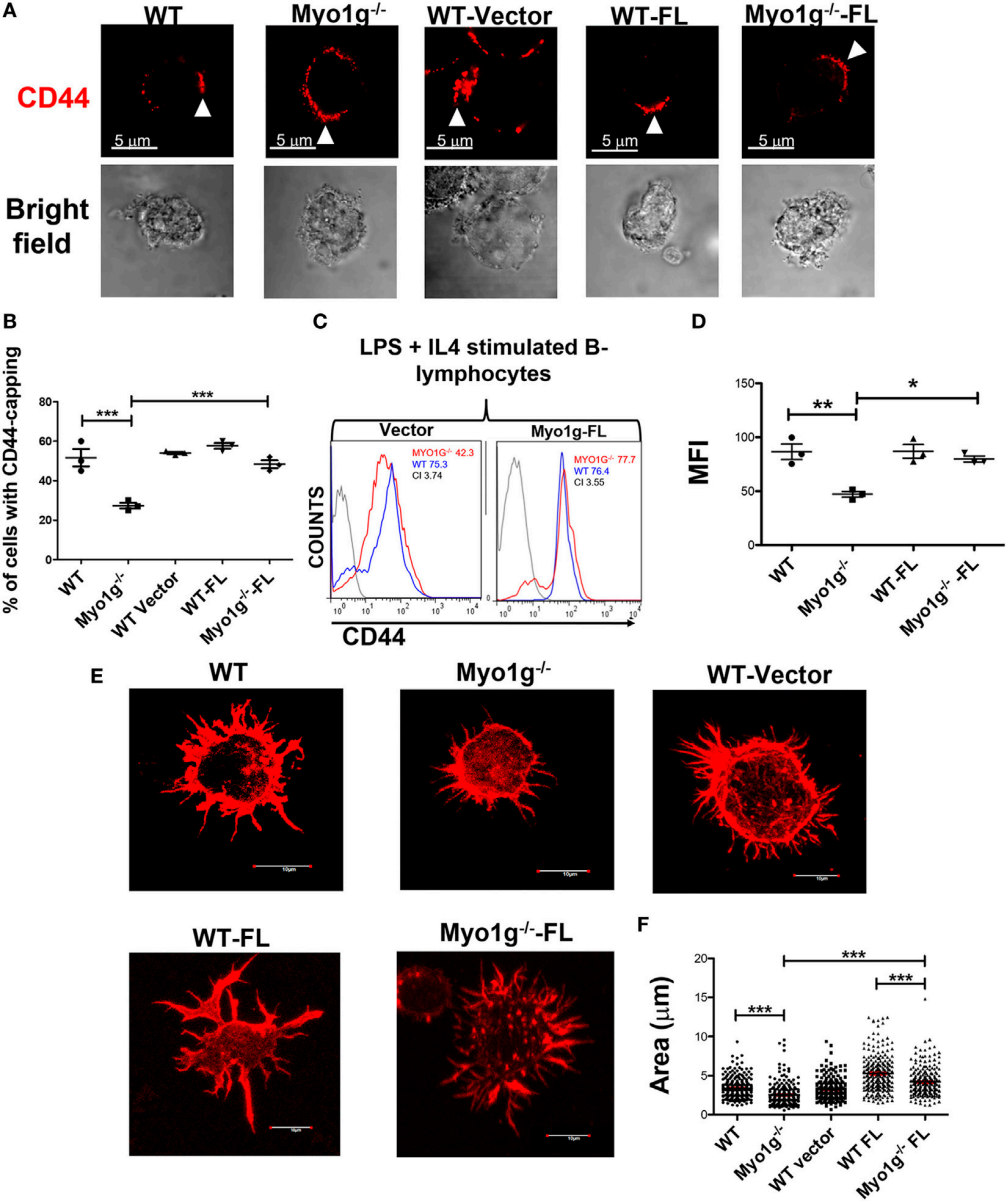
Transfection with full-length Myo1g rescue CD44-dependent capping and spreading in Myo1g-deficient B-cells. **(A)** Confocal images of LPS plus IL4-activated B cells from WT, Myo1g^−/−^, WT, or Myo1g^−/−^ transfected with an empty vector or Myo1g-FL. Arrows illustrate the localization of CD44 in B lymphocytes (scale bar 5 µm). **(B)** Percentage of cells with CD44-capping. Images of randomly selected files were taken and the percentage of B lymphocytes with capping was calculated. A total of 200 cells per data set from three independent experiment were analyzed. One-way ANOVA test was used in these experiments, values are mean ± SD (***P < 0.001) (*n* = 3). **(C)** Representative expression of CD44 in LPS plus IL4-activated B cells transfected or not with Myo1g-FL from WT or Myo1g-deficient B lymphocytes, the graph shows the expression of CD44 in B lymphocytes (10,000 events in a gate of B220 + B cells). **(D)** Expression of CD44 in LPS plus IL4-activated B cells transfected or not with Myo1g-FL from WT or Myo1g-deficient B lymphocytes. One-way ANOVA test was used in these experiments, values are mean ± SD (* *P* < 0.05, **P < 0.01) (*n* = 3). **(E)** Confocal images of activated B cells from WT or Myo1g-deficient mice over NIMR8. **(F)** The area of spread B lymphocytes was evaluated, a total of 150 cells per data set from three independent experiments were analyzed. One-way ANOVA test was used in these experiments, values are mean ± SD (***P < 0.001).

In these reconstituted B-lymphocytes, we analyzed CD44-expression, CD44-induced capping and spreading. As shown in Figures 9A,B, we observed the formation of CD44-cap structures in Myo1g-reconstituted B-lymphocytes. CD44-expression was also restored to WT levels when Myo1g expression was reestablished (Figures 9C,D). An increased number and size of membrane projections was also observed in Myo1g-reconstituted B-lymphocytes (Figure 9E). To further validate this result, we measured cell-area of these lymphocytes, the data illustrate a recovery in their area (Figure 9F). These data support the participation of Myo1g in the recycling of CD44, and its presence is needed to maintain membrane projections in B lymphocytes.

### Absence of Myo1g Affects CD44-Dependent Adhesion, Uptake of HA-Coated Beads, and Migration in B Lymphocytes

As previously mentioned, CD44 is involved in adhesion, migration (88, 89), and phagocytosis (43), among other functions. Thus, CD44 adhesion of Myo1g^−/−^ B lymphocytes was evaluated. It was observed that Myo1g^−/−^ B cells adhered less efficiently than WT B cells to HA. To demonstrate specificity, we pretreated B lymphocytes with α–CD44 clone IM7 (who interferes with the CD44-HA interaction), this pretreatment altered the adhesion to HA of WT and Myo1g^−/−^B cells. B cells from either Myo1g^−/−^ or WT B cells did not show defects in IgM-adhesion (Figure S11A in Supplementary Material).

CD44-dependent endocytosis was also evaluated. Thus, we treated micro-spheres with HA, then, these beads were incubated with B lymphocytes from WT or Myo1g-deficient mice; in some experiments, B cells were pretreated with α-CD44 clone IM7. The results indicated reduced uptake of HA-coated beads. The differences disappeared by IM7 pretreatment, indicating a CD44-dependent mechanism (Figure S11B in Supplementary Material).

**Figure 10:**
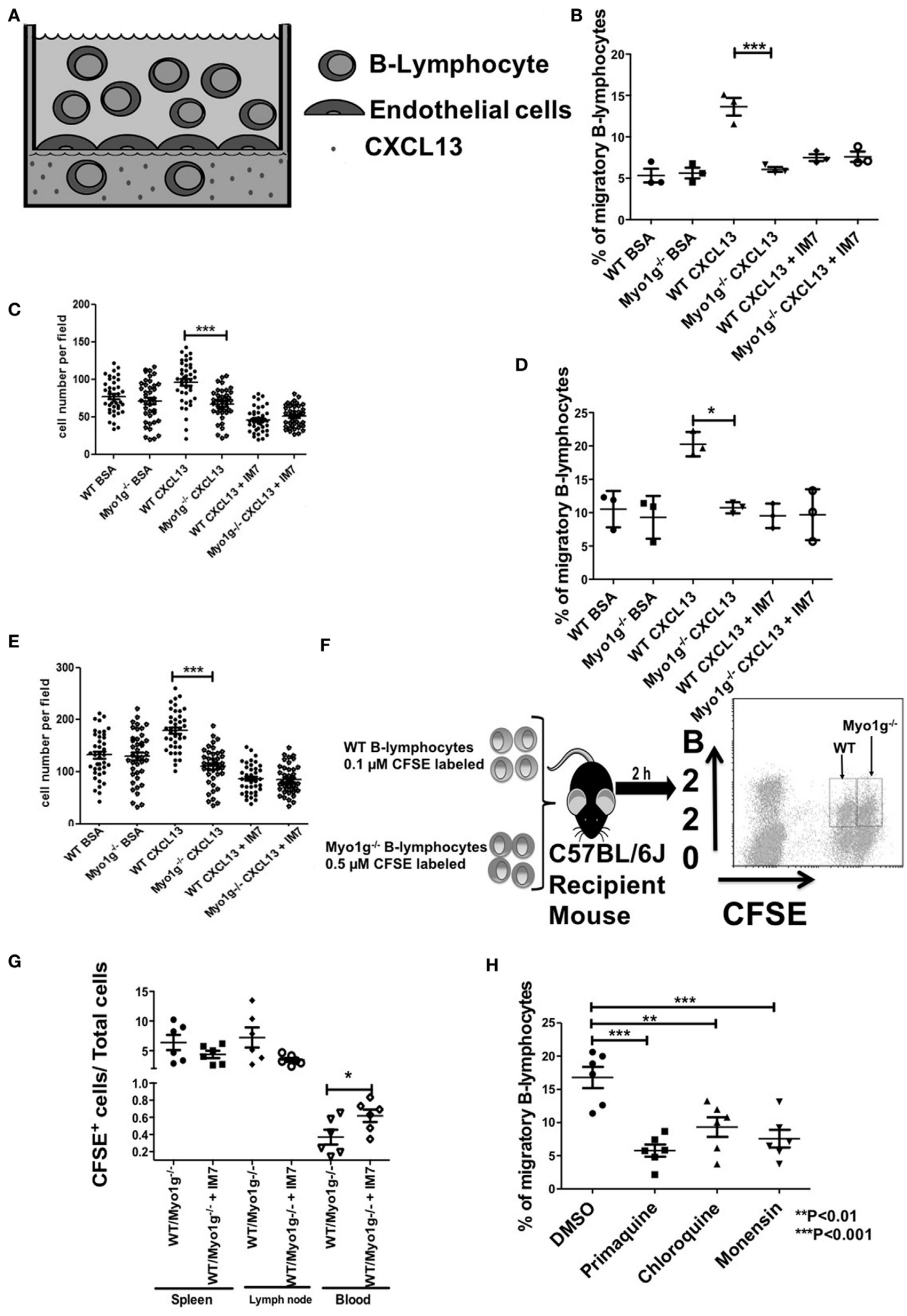

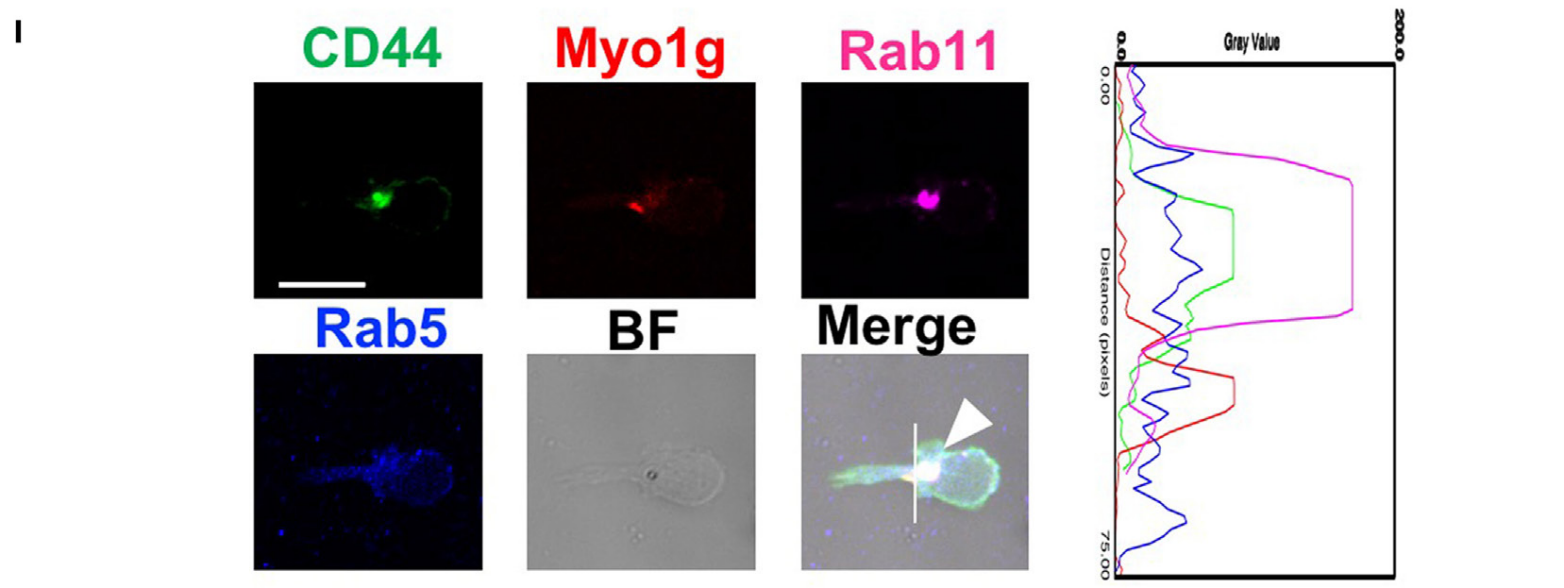
Myo1g deficiency affects the migration of B lymphocytes. **(A)** Scheme of transendothelial migration where 4 × 10^5^ B cells were placed in the upper compartment. **(B,C)** Analysis from resting WT or Myo1g-deficient B-lymphocytes. **(D,E)** Analysis of LPS plus IL-4 activated WT or Myo1g-deficient B lymphocytes. **(B,E)** Show the CXCL13-induced migration of B cells. **(C,E)** Percentage of migratory B cells that crossed the endothelial barrier. **(F)** Scheme of *in vivo* homing assay one-way ANOVA tests were used in these experiments, values are mean ± SD (*P < 0.05, ***P < 0.001) (*n* = 3). **(G)** 1 × 10^7^ cells were transferred to recipient mice, the graph illustrates B cell recovery (CFSE^+^) percentage (WT/Myo1g^−/−^) from spleen, lymphatic nodes, and blood from Myo1g^−/−^ and WT mice. Data are mean ± SD from three mice per assay in three independent experiments. One-way ANOVA test was used in these experiments, values are mean ± SD (*P < 0.05) (*n* = 6). **(H)** 4 × 10^5^ resting B lymphocytes treated with vehicle, primaquine, chloroquine, or monensin were placed in the upper compartment during transendothelial transmigration. **(I)** Resting B-cells were placed on fibronectin-covered coverslips for 30 min, after that, the cells were treated with CXCL13 and incubated for 20 min, and then fixed and stained with antibodies for Rab5, Rab11, Myo1g, and CD44 for 20 min; the arrows indicate the location of these molecules in migratory B cells. The cells were then analyzed by confocal microscopy, scale bar 5 µm.

CD44-dependent migration was then evaluated in more physiological settings, using transendothelial cell migration and *in vivo* homing assays. These methods are illustrated in Figures 10A,F, respectively. In some cases, we performed the same pretreatment with α-CD44 clone IM7 to evaluate the role of CD44 in this physiologic process. In the transendothelial migration assays, the Myo1g-deficient B cells demonstrated reduced migration in response to a CXCL13 gradient, compared with WT B lymphocytes. Pretreatment with IM7 antibody reduced the migration of WT B cells (Figure 10B), demonstrating the role of CD44 in this process. Simultaneously, the number of cells per field, in the filter containing the endothelial monolayer, was quantified. Less Myo1g^−/−^ B cells adhered to the monolayer, and the adhesion was CD44-dependent as was shown by the inhibition observed with the IM7 antibody (Figure 10C). The experiments described above were performed using resting B cells. We, therefore, repeated these studies using LPS plus IL-4 activated B cells, the results become like those observed with resting B cells (Figures 10D,E).

As described above, we then measured migration *in vivo*, by inoculating mixtures of CFSE-labeled B cells from WT or Myo1g-deficient mice to a WT recipient mouse (iv route) and then, we quantified the migrating cells in spleen, lymph nodes, and peripheral blood (Figure 10F). The results obtained from these experiments showed a reduced number of Myo1g^−/−^ B cells in the spleen and the lymph nodes. Pretreatment with α-CD44 clone IM7, as shown *in vitro*, caused the reduction of recruitment to spleen and lymph nodes, with a corresponding greater proportion of B cells in the peripheral blood (Figure 10G). Taken together, these results indicate that Myo1g-deficient B cells have an impaired ability to cross the endothelial barrier.

The recycling of CD44 and lipid rafts is crucial in diverse cell processes, for this reason, we used primaquine-, chloroquine-, and monensin-treated B lymphocytes to examine: transendothelial migration (Figure 10H); 2D migration under CXCL12 and CXCL13 (Figures S12A–C in Supplementary Material); spreading (Supplementary Figures S12D,E in Supplementary Material); or surface expression of CD44 after HA treatment (Figure S12F in Supplementary Material). We registered an impaired transendothelial migration in recycling by all the inhibitors used in this assay (Figure 10H). Likewise, we observed a reduction in the speed, accumulated and Euclidian distance and straightness by the drugs (Figures S12A–C in Supplementary Material). We also detected the absence of membrane protrusions under these conditions (Figures S12D,E in Supplementary Material). Finally, the presence of CD44 on the surface of primaquine-treated cells was reduced (Figure S12F in Supplementary Material). To further corroborate these results, we evaluated the location of CD44, Myo1g, Rab5, and Rab11 in migrating B lymphocytes. As it can be seen in Figure 10I, we found a spatiotemporal organization of these four molecules in migratory cells. As a whole, these results support a model of interaction between these four molecules during migration.

## Discussion

In this study, we established that Myo1g drives the mobilization of CD44. B cells lacking Myo1g revealed defective mobilization of CD44 and lipid rafts. Collectively, our data demonstrate a role for Myo1g in the traffic of lipid raft-dependent molecules.

Confocal microscopy studies showed that the capping of CD44 in resting B cells was unchanged in the absence of Myo1g. This suggests that Myo1g is not required for the mobilization of this protein under steady-state conditions. Alternatively, Myo1g may be redundant with other proteins, including class I myosins (Myo1c or Myo1e). In contrast, LPS plus IL4-activated B cells demonstrated impaired CD44 polarization. This is likely due to ineffective CD44 mobilization inside the cell, which may not be completely compensated by Myo1c or Myo1e. These data suggest an important role for Myo1g in the traffic of CD44. Previous studies have shown the participation of Myo1c in the traffic of CD55, Glut4, and VEFGR2 in HeLa cells (85), adipocytes (90), and endothelial cells (91), respectively. Similarly, this study demonstrates a role for Myo1g during CD44 translocation in activated B cells.

During cell migration, the role of regulators of actin dynamics, including actin bundles and capping proteins, are expected to play a critical role in controlling the architecture and dynamics of the actin meshwork that propels non-adhesive B cell migration. The deficiency in the polarization of CD44 in Myo1g-deficient B cells affected cell migration. Consistent with a previous study from our group, Myo1g^−/−^ B cells demonstrated slower migration trajectories than WT B cells (33). One possible cause for the reduced mobility of Myo1g-deficient B cells is the delay in the traffic of adhesion molecules, which includes CD44. Thus, the deficiency of Myo1g affects the traffic of these molecules. The entry of CD44 and integrins into cholesterol and glycosphingolipid-rich domains was suggested as a mechanism of cell migration initiation since adhesion molecules and lipid rafts carry caveolin, thus enabling initiation of the recycling process. Moreover, cytoskeletal proteins and GTPases are simultaneously recruited to these recycling vesicles.

Previous biochemical and ultrastructural studies have demonstrated that CD44 is present in both raft and non-raft regions of cell membranes, similar to the motor protein Myo1g in B cells (33).

The loss of function of Myo1g significantly delayed CD44-cell spreading and plasma membrane delivery of raft-associated molecules in B cells. Expression of Myo1g aided the localization of lipid rafts in LPS plus IL4 activated B cells. This is consistent with observations by Bransdtatter et al. in 2012, showing that when Myo1c is overexpressed, the expression of several markers of lipid rafts, such as CD55 and CD59, increases in the plasma membrane (85).

CD44 is dynamic, and its levels are maintained by internalization, recycling, and delivery to the plasma membrane. We have previously shown that in B cells, Myo1g and GM1 co-localize at the plasma membrane (33). LPS plus IL4 stimulated B cells internalize and recycle CD44. This is also seen in Myo1g-deficient B cells, but there, the recycling process is not as efficient, leading to increased accumulation of CD44 inside the cell with consequent reduction in surface expression. The decrease in CD44 may affect formation of the immunological synapse in B cells as has been observed in T cells where the deficiency of CD44 affected formation of the immunological synapse between T cells and dendritic cells (92).

CD44 is mobilized to caveolae and is subsequently endocytosed (84, 93). Caveolae is a specialized structure that contains various associated molecules or microdomains such as lipid rafts (84, 94). CD44 has been found in these membrane microdomains (93, 95, 96), with various roles in cell adhesion and migration (96). The composition, plasticity, and surface area of the plasma membrane is controlled by specialized endocytic recycling pathways, which maintain the balance between the endocytosis and exocytosis of specific proteins and lipids. Cargo proteins associated with lipid raft membranes are internalized by clathrin-independent endocytosis. However, the molecular determinants that regulate intracellular trafficking in this pathway have yet to be established (97).

We also observed that deficiency of Myo1g affects the expression of lipid rafts on the surface of B cells. Interestingly, an association of Myo1g with lipid raft microdomains was first indicated by proteomics studies, which identified this myosin in purified lipid raft fractions isolated from B cells (98). The decrease in lipid rafts may affect the clustering of signaling molecules, with consequences for several process such as cell migration (99), BCR signaling (100), and dissociation of the CD3-CD8 complex in T cells (101). In fact, disruption of lipid rafts by nystatin and methyl-β-cyclodextrin affects the capping of IgM (102). A recent study demonstrated the presence of Myo1g in a complex of cytosolic proteins, which include the GTPase RalA and the GTPase-activating protein srGAP2 (34). RalA has been associated with the exocytosis of integrins in mouse embryonic fibroblasts (7), and srGAP2 has been linked to migration and adhesion of neuronal cells (103).

In the same context, adhesion molecules can activate the GTPase RalA and RalA-specific GEF. This GTPase can interact with the exocyst subunit Sec5. This subunit is required for cell spreading and raft exocytosis after cell adhesion. Downregulation of sec5 produces a reduction in cell spreading and cell adhesion (104). The interaction between the exocyst complex and the GTPase CDC42 is necessary for polarized secretion in yeast (104). This phenomenon has been linked to srGAP2 by through the inactivation of CDC42 in neuronal cells during cell migration (105). The presence of srGAP2 at the phagocytic cup in HEK293T cells has also been described (105). These observations are consistent with our previously published study in which the presence of Myo1g was described in those sites (105), as well as with results from this study showing that Myo1g actively participates in the recycling process of CD44. We believe that part of the defects in the recycling process of CD44 in the Myo1g-deficient B cells can be attributed to the signaling molecules arriving at different times, because we did not detect major defects in the function of these cells. We are currently working to determine the mechanisms of this delay, but we believe that a delay in assembling this molecular complex may represent the best explanation for our observations.

Taken together, these findings demonstrate the defects in CD44-dependent functions in Myo1g-deficient B cells. We observed reduction in all the functions examined (migration, adhesion, and phagocytosis). Our results correlate with the work of Cairns et al. (106) in which the authors observed an impaired clearance of apoptotic neutrophils by monocytes with reduced expression of CD44 (106), defective migration of CD44 KO fibroblasts after treatment with TGF-β (107), or through the use of α-CD44 antibodies, which block leukocyte migration in the skin (108). This study shows that Myo1g is a predominant regulator of CD44 recycling in B cells and is important in CD44-dependent cell functions.

## Ethics Statement

The mice were produced at the Centro de Investigación y de Estudios Avanzados (Mexico City, Mexico) animal facility. The Animal Care and Use Committee of Centro de Investigación y de Estudios Avanzados approved all experiments.

## Author Contributions

OL-O designed and performed the experiments; and wrote the paper. LS-A designed the experiments, supervised the work, and wrote the paper.

## Conflict of Interest Statement

The authors declare that the research was conducted in the absence of any commercial or financial relationships that could be construed as a potential conflict of interest.
