# Supplementary material for: Myosin 1g Contributes to CD44 Adhesion Protein and Lipid Rafts Recycling and Controls CD44 Capping and Cell Migration in B Lymphocytes
Source: Front Immunol. 2017 Dec 11;8:1731. doi: 10.3389/fimmu.2017.01731 (PMC5732150; doi:10.3389/fimmu.2017.01731)
Supplement: Supplementary file 1 [file Data_Sheet_1.pdf]

Supplementary Material

Myosin 1g contributes to CD44 adhesion protein and lipid rafts recycling and controls CD44 capping and cell migration in B lymphocytes.

López-Ortega O[1], Santos-Argumedo L[1*].

1. Departamento de Biomedicina Molecular, Centro de Investigación y de Estudios Avanzados del Instituto Politécnico Nacional, Ciudad de México, México.

* Corresponding Author: Dr. Leopoldo Santos-Argumedo; E-mail: lesantos@cinvestav.mx; Telephone: (+52-55)-57473800 Ext. 3323, Fax: (+52-55)-57473938. Departamento de Biomedicina Molecular, Centro de Investigación y de Estudios Avanzados del IPN, Avenida IPN 2508, CP 07360, D.F., México.

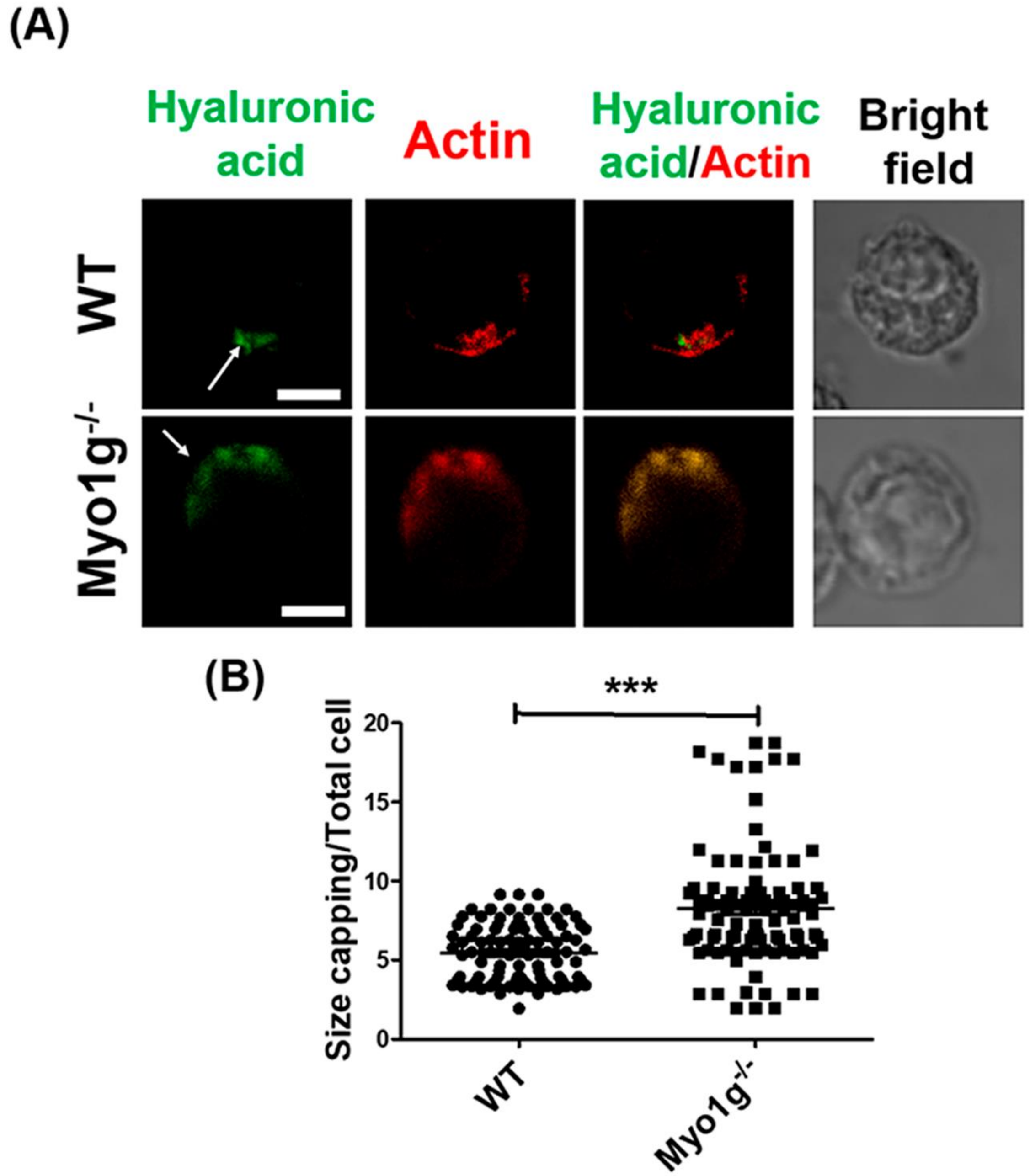

Supplementary figure 1. Myo1g-deficient B cells exhibit impaired CD44-capping. A) Confocal images from WT or Myo1g$^{-/-}$ LPS plus IL4-activated B cells. Arrows indicate the localization of CD44 in B cells after polarization (scale bar 5 μm). B) Size of capping: images of randomly selected files were taken and B lymphocytes showing capping were quantified. A total of 100 cells were analyzed in three independent experiment; Student´s t-test was used in this graph, values are mean ± SD, (***P<0.001).

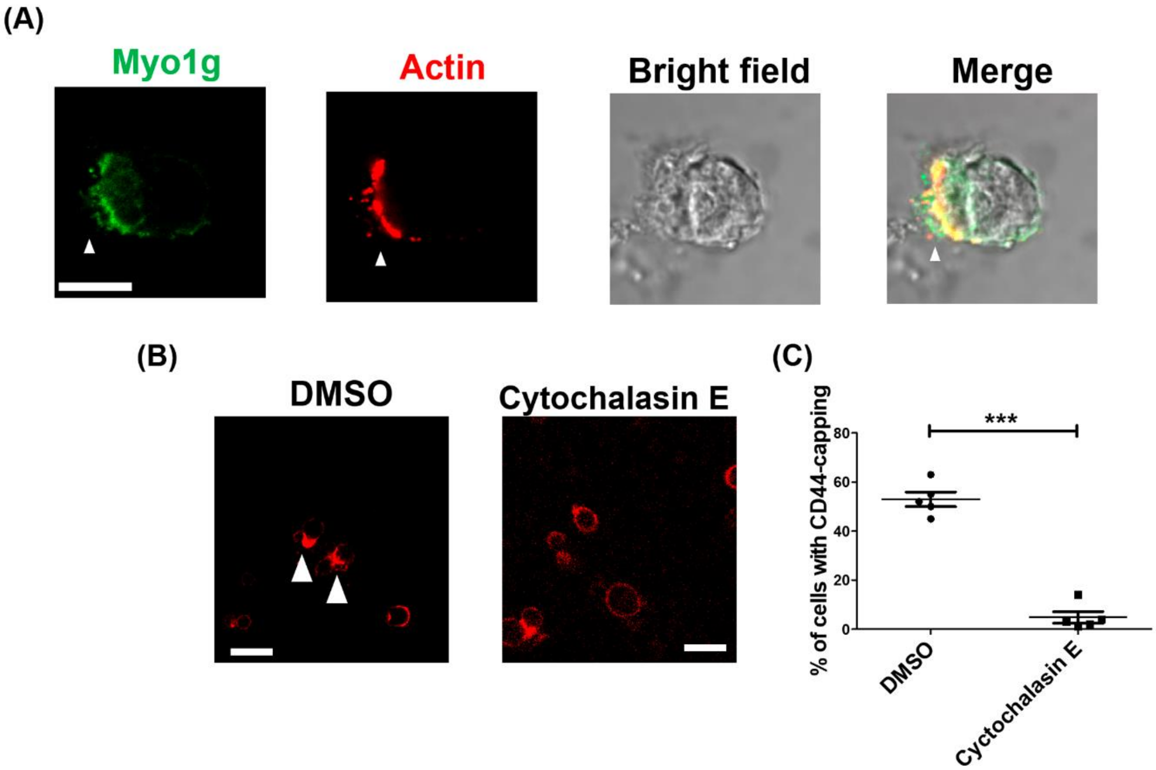

Supplementary figure 2. Actin-cytoskeleton is necessary for CD44-polarization. A) Confocal images of LPS plus IL4-activated WT B cells. Arrows indicate the localization of Myo1g and actin in B lymphocytes (scale bar 5 μm) (A total of 150 cells were analyzed in three independent experiments). B) Confocal images of LPS plus IL4-activated WT treated or no with Cytochalasin E. Arrows indicate the localization of CD44 in B lymphocytes (scale bar 5 μm) (A total of 100 cells were analyzed in three independent experiments). C) Percentage of CD44-capping: images of randomly selected files were taken and the percentage of B lymphocytes with capping was calculated. A total of 100 cells were analyzed in three independent experiment; Student´s t-test was used in this graph, values are mean ± SD, (***P<0.001).

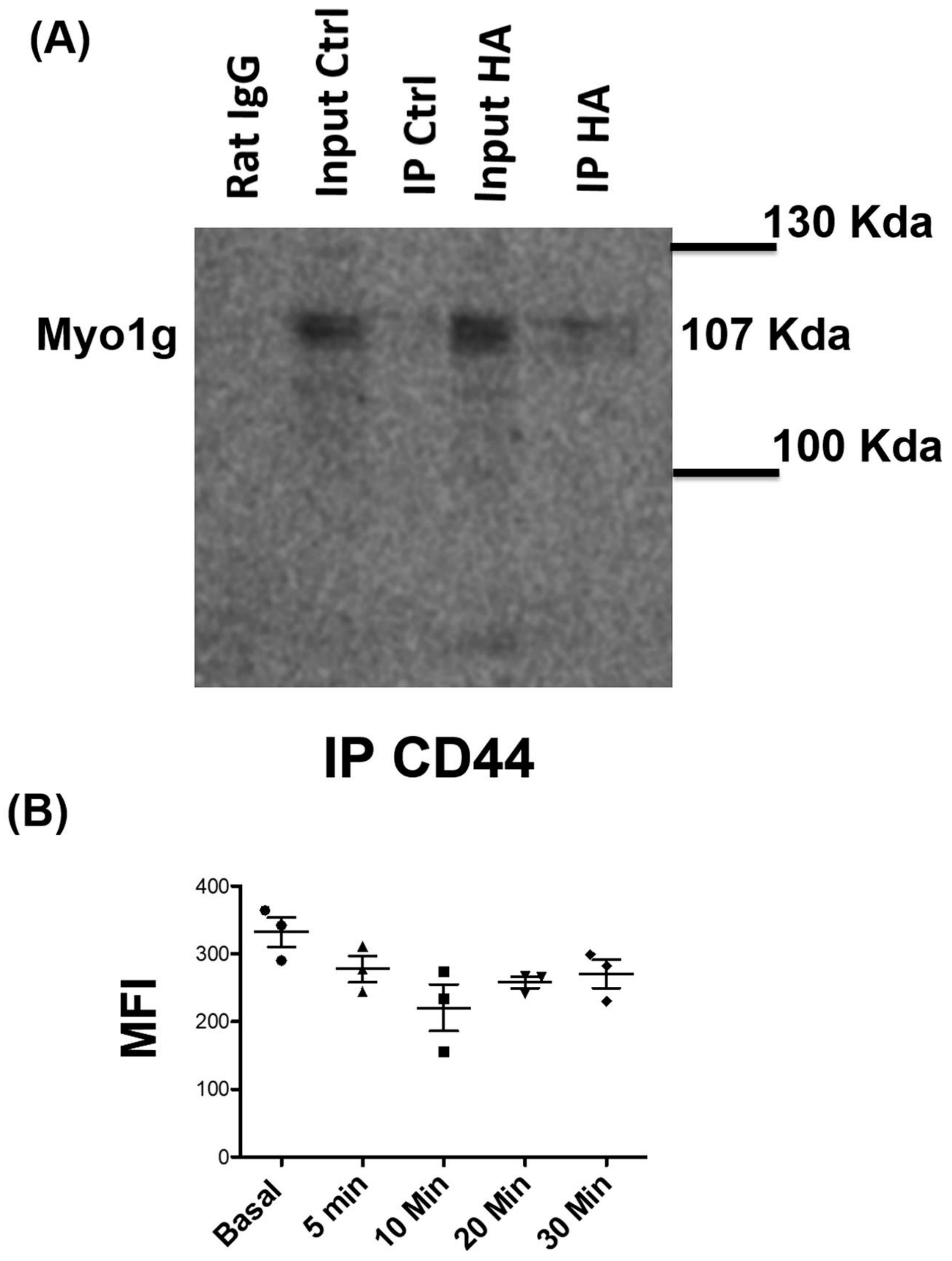

(A)
Rat IgG
Input Ctrl
IP Ctrl
Input HA
IP HA
130 Kda
Myo1g
107 Kda
100 Kda
IP CD44
(B)
MFI
400
300
200
100
0
Basal
5 min
10 Min
20 Min
30 Min

Supplementary figure 3. CD44 interact with Myo1g after HA-Treatment in B lymphocytes. Immunoprecipitation with α-CD44 (NIMR8) α-Rat (as isotype control) of LPS plus IL4-activated B cells treated with hyaluronic acid. B) Expression of CD44 in HA-treated B cells was evaluated at different time points. One-way ANOVA test was used in these experiments, values are mean ± SD, (n = 3).

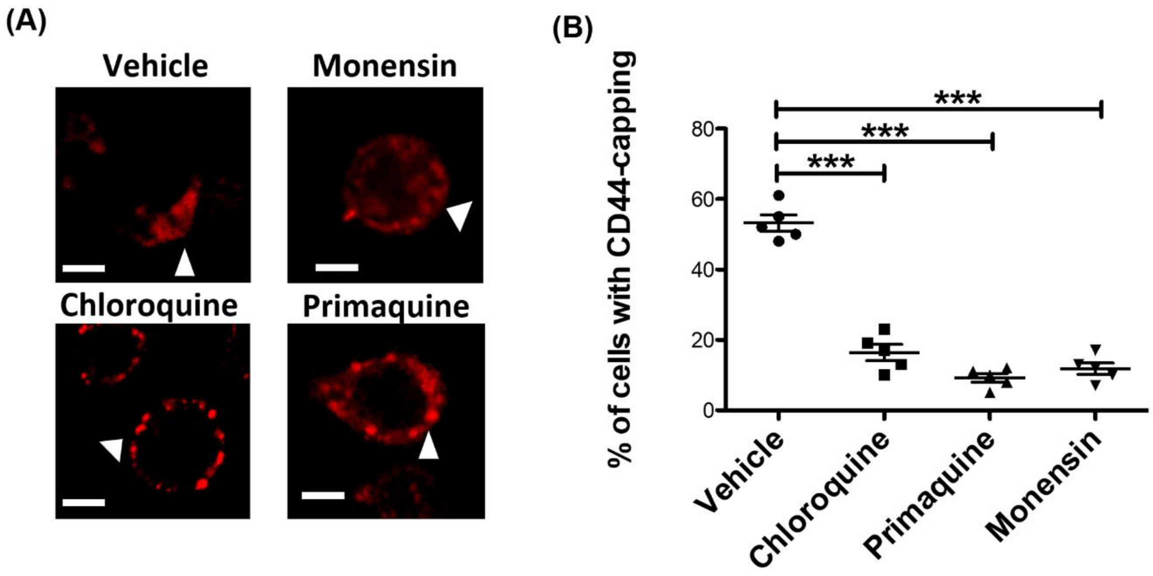

Supplementary figure 4. Recycling is required for CD44-capping. A) Confocal images of LPS plus IL-4 activated B lymphocytes treated with vehicle, primaquine, chloroquine or monensin. Arrows indicate the localization of CD44 on the plasma membrane of B lymphocytes (scale bar 5µm). B) Percentage of cells with CD44-capping: images of randomly selected files were taken and the percentage of B lymphocytes with capping was calculated. One-way ANOVA test was used in this graph values are mean ± SD, (***P<0.001) (150 cells per data set, 30 cells per each experiment n = 5).

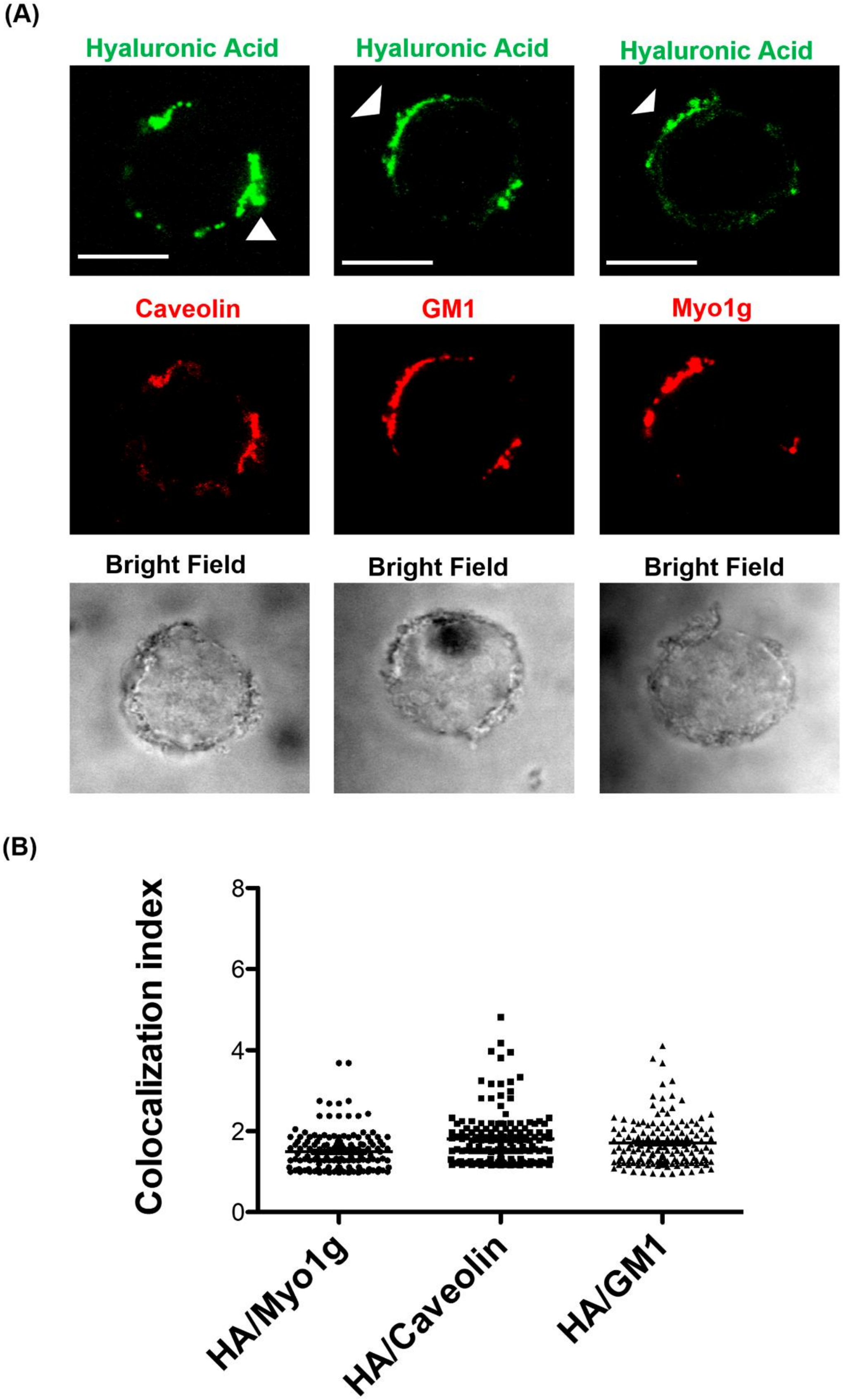

(A)
Hyaluronic Acid
Hyaluronic Acid
Hyaluronic Acid
Caveolin
GM1
Myo1g
Bright Field
Bright Field
Bright Field
(B)
Colocalization index
8
6
4
2
0
HA/Myo1g
HA/Caveolin
HA/GM1

Supplementary figure 5. Hyaluronic acid drives the polarization of Myo1g, caveolin and lipid rafts. Confocal images from WT LPS plus IL4-activated B cells. Arrows indicate the localization of Hyaluronic acid (Green) and caveolin, GM1 or Myo1g (Red) in B cells after polarization (scale bar 5 μm). B) Polarization coefficient of caveolin, GM1 and Myo1g with hyaluronic acid (HA) derived from the analysis of a total of 150 cells with capping in three independent experiments, values are mean ± SD.

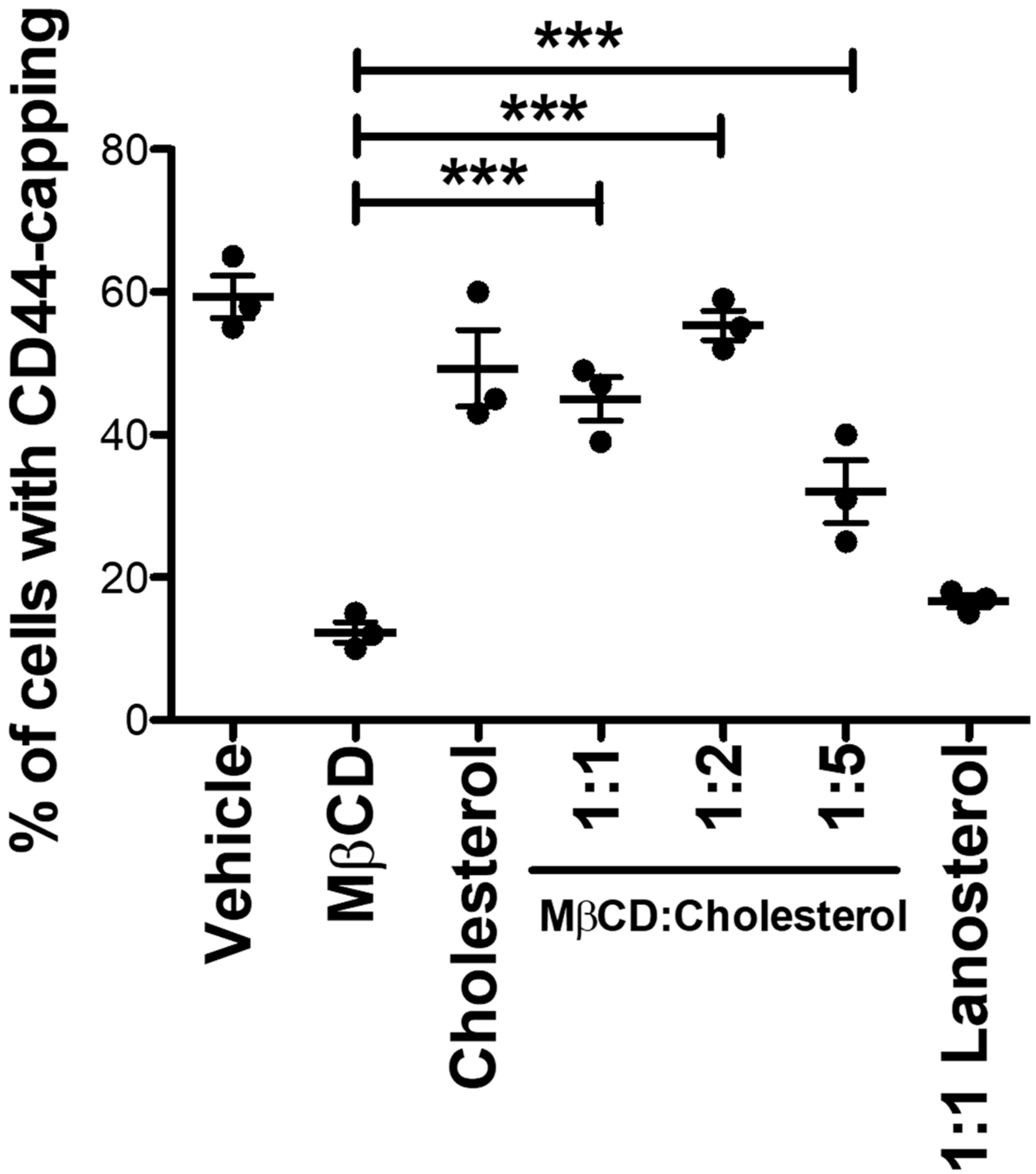

Supplementary figure 6. Cholesterol is necessary for CD44-polarization in B lymphocytes. LPS plus IL-4 activated B lymphocytes were treated with MβCD (5 mM) in combination with different concentrations of cholesterol (1μg/ml, 2μg/ml and 5μg/ml) or lanosterol (1μg/ml). One-way ANOVA test was used in this graph values are mean ± SD, (*P<0.05, ***P<0.001) (A total of 150 cells were analyzed in three independent experiments).

(A)

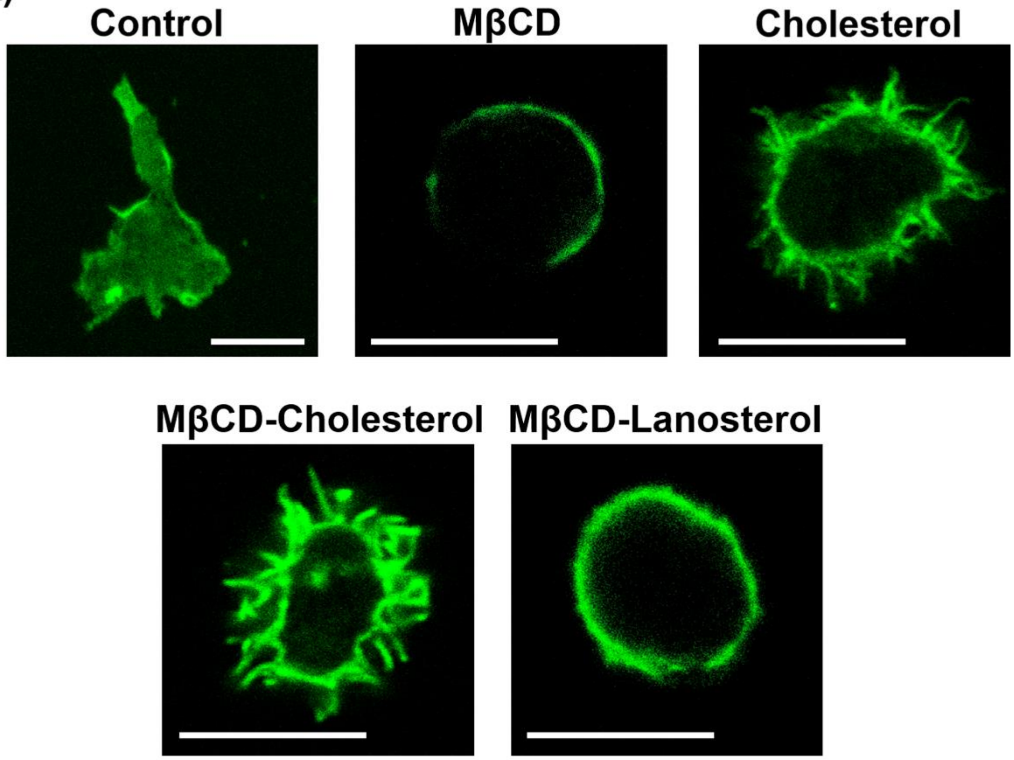
Control
MβCD
Cholesterol
MβCD-Cholesterol
MβCD-Lanosterol

(B)

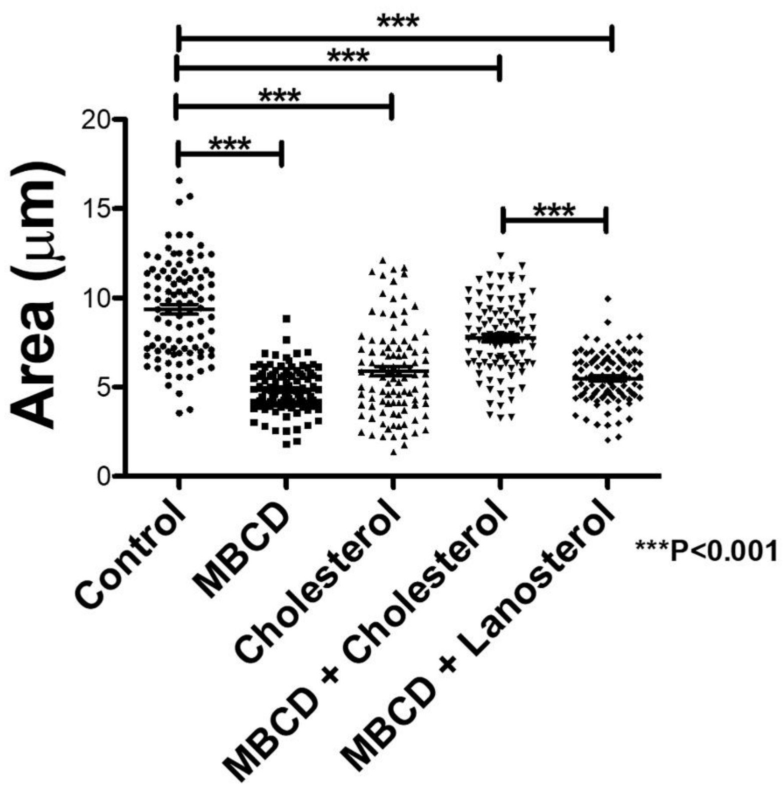
***
***
***
***
***
20
15
10
5
0
Area (μm)
Control
MBCD
Cholesterol
MBCD + Cholesterol
MBCD + Lanosterol
***P<0.001

Supplementary figure 7. Cholesterol is necessary for CD44-spreading in B lymphocytes. Confocal images of spreading over NIMR8 of activated B cells treated with with MβCD (5 mM) in combination with different concentrations of cholesterol (1μg/ml) or lanosterol (1μg/ml). "Spread" B lymphocytes were evaluated for area in B), Area from spread B lymphocytes, a total of 100 cells per data set from three independent experiment were analyzed. One-way ANOVA test was used in these experiments, values are mean ± SD (***P<0.001).

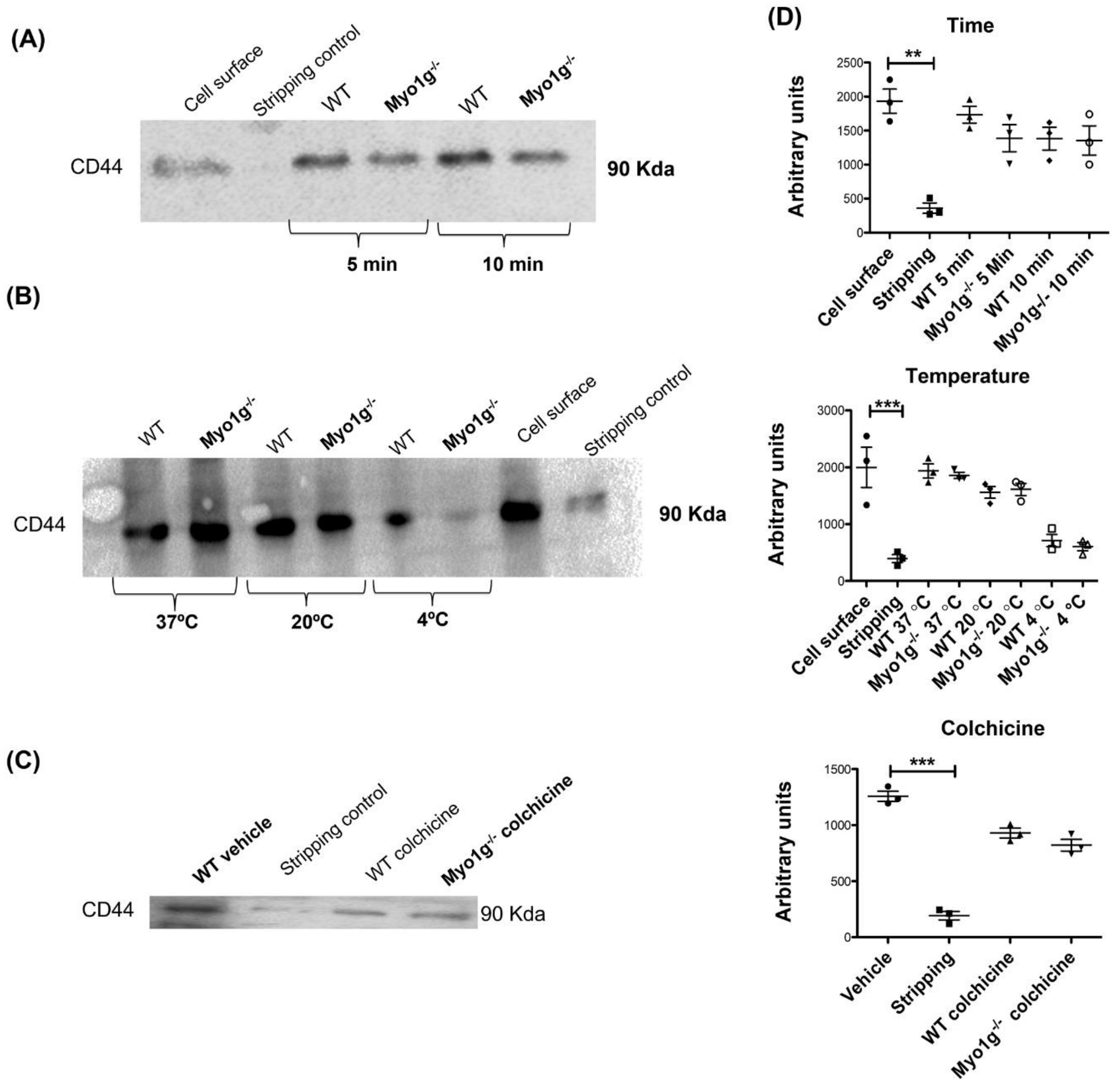

Supplementary figure 8. Internalization of CD44 is similar in WT and Myo1g$^{-/-}$ B cells. (A) Activated and biotinylated B lymphocytes belonging of WT or Myo1g$^{-/-}$ mice were incubated 5 or 10 minutes with NIM-R8 mAb to induce CD44 internalization. (B) In a similar set of

experiments the cells were incubated 5 minutes at 37 ºC, 20 ºC or 4 ºC. (C) Alternatively, the cells were pretreated with vehicle or colchicine during 30 minutes before the induction of 5 minute CD44 internalization with NIM-R8 mAb. In all cases, the cells were trypsinized and then incubated with unlabeled streptavidin; afterward, these cells were lysed with RIPA buffer and precipitated with streptavidin-agarose, loaded in 12% SDS-PAGE, transferred to nitrocellulose membrane and revealed with KM-201 anti-CD44 mAb. D) Densitometry analysis of internalized CD44 in WT or Myo1g$^{-/-}$ activated B cells. Images were analyzed using the ImageJ software. One-way ANOVA test was used to analyze three independent experiments, values are mean ± SD, (**P<0.01), (***P<0.001). The stripping control correspond to cells treated with trypsin and unlabeled streptavidin without internalization to register remaining CD44 on the surface.

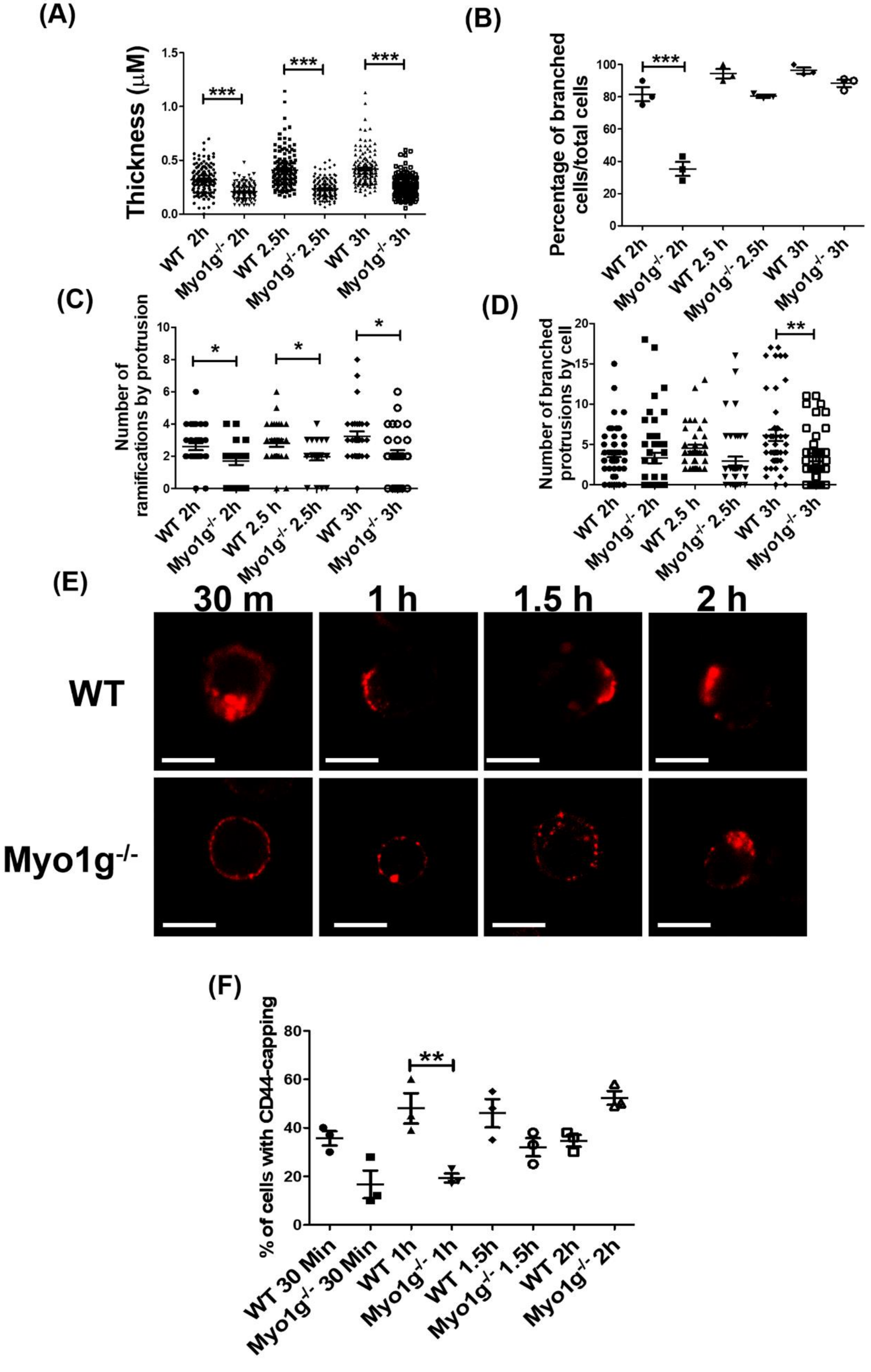
(A)
Thickness (μM)
1.5
1.0
0.5
0.0
***
***
***
WT 2h
Myo1g-/- 2h
WT 2.5h
Myo1g-/- 2.5h
WT 3h
Myo1g-/- 3h
(B)
Percentage of branched cells/total cells
100
80
60
40
20
0
***
WT 2h
Myo1g-/- 2h
WT 2.5 h
Myo1g-/- 2.5h
WT 3h
Myo1g-/- 3h
(C)
Number of ramifications by protrusion
10
8
6
4
2
0
*
*
*
WT 2h
Myo1g-/- 2h
WT 2.5 h
Myo1g-/- 2.5h
WT 3h
Myo1g-/- 3h
(D)
Number of branched protrusions by cell
20
15
10
5
0
**
WT 2h
Myo1g-/- 2h
WT 2.5 h
Myo1g-/- 2.5h
WT 3h
Myo1g-/- 3h
(E)
30 m
1 h
1.5 h
2 h
WT
Myo1g-/-
(F)
% of cells with CD44-capping
80
60
40
20
0
**
WT 30 Min
Myo1g-/- 30 Min
WT 1h
Myo1g-/- 1h
WT 1.5h
Myo1g-/- 1.5h
WT 2h
Myo1g-/- 2h

Supplementary figure 9. The absence of Myo1g affects membrane projections in B cells. Confocal images of activated B cells from WT or Myo1g-deficient mice. "Spread" B lymphocytes were evaluated at different time points for: A) thickness of projections B) percentage of cells with branched projections C) number of ramifications by projection and D) the number of branched protrusions per cell. A total of 100 cells were analyzed in three independent experiment; One-way ANOVA tests were used in these graphs, values are mean ± SD (**$P<0.01$, ***$P<0.001$). E) Confocal images of the CD44-capping of WT or Myo1g-deficient B cells. F) Percentage of cells with CD44-capping. Images of randomly selected files were taken and the percentage of B lymphocytes with capping was recorded. A total of 200 cells were analyzed in three independent experiments; One-way ANOVA test was used in this graph, values are mean ± SD, (**$P<0.01$).

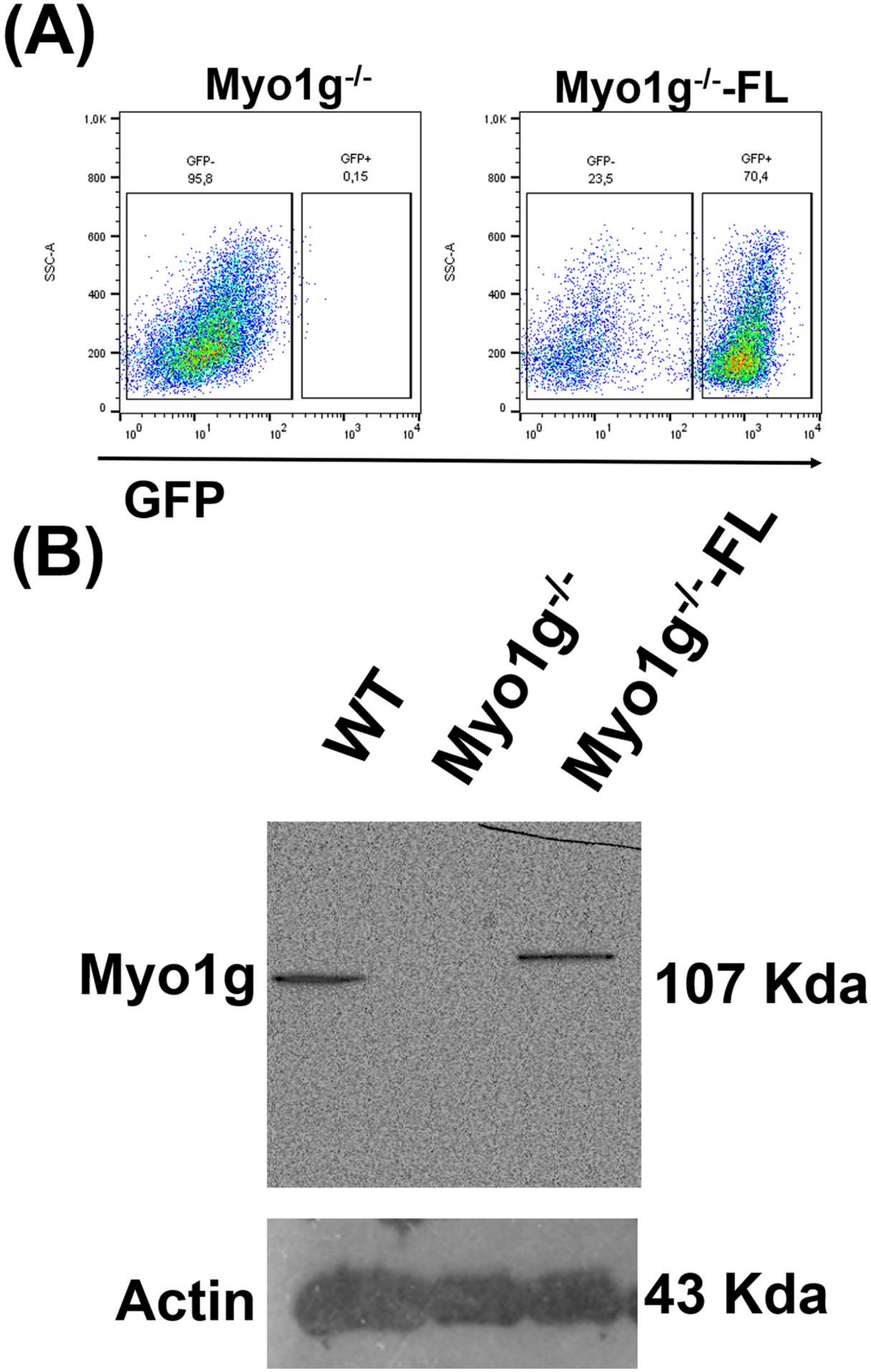
(A)
Myo1g-/-
Myo1g-/--FL
1,0K
800
600
400
200
0
SSC-A
GFP-
95,8
GFP+
0,15
GFP-
23,5
GFP+
70,4
GFP
(B)
WT
Myo1g-/-
Myo1g-/--FL
Myo1g
107 Kda
Actin
43 Kda

Supplementary figure 10. Recovery of expression of Myo1g in Myo1g-deficient B lymphocytes. A) Dot plots of transfected Myo1g-FL in Myo1g$^{-/-}$ B cells. B) Immunodetection of Myo1g in WT, Myo1g$^{-/-}$, and Myo1g$^{-/-}$ transfected with Myo1g-FL B lymphocytes.

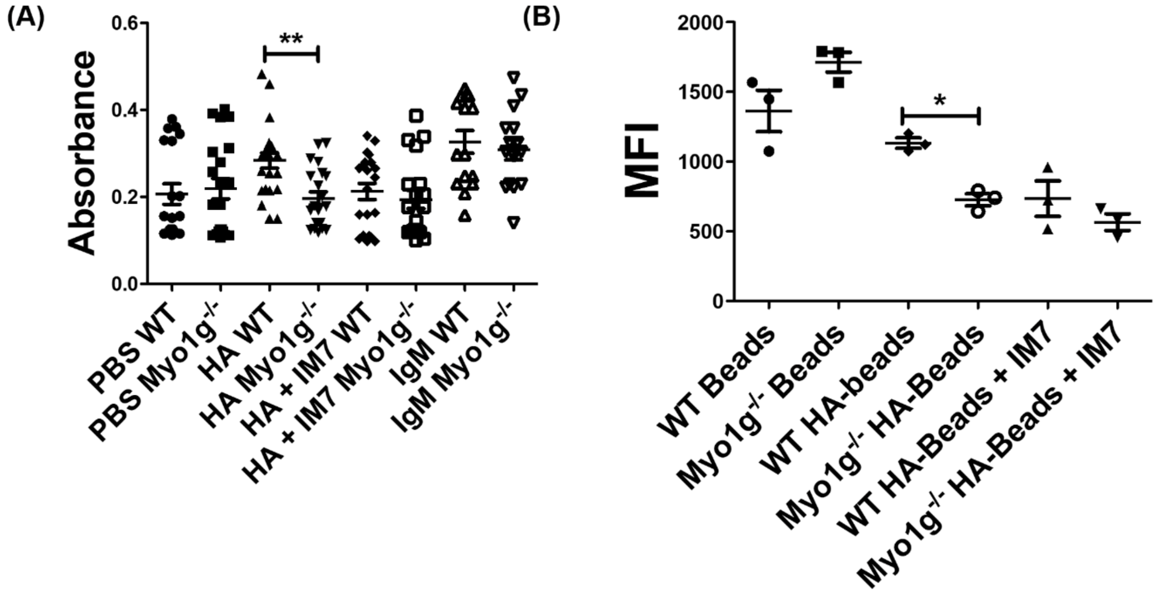

Supplementary figure 11. The absence of Myo1g affects adhesion and bead-uptake in B lymphocytes. A) Cell-adhesion of LPS plus IL-4 activated WT or Myo1g$^{-/-}$ B lymphocytes to HA or α-IgM coated plates. Cell adhesion was registered by the amount of crystal violet remaining in the plates (recorded at 540 nm) after extensive washing. The absorbance was determined in at least four wells per condition, in seven independent experiments. One-way ANOVA test was used in this graph, values are mean ± SD, (**$P<0.01$). B) B-cells from Myo1g$^{-/-}$ or WT mice were incubated with HA coated 0.5 µm polystyrene fluorescent microspheres at bead-to-cell ratio of 10:1. The cells were then harvested and washed with PBS. Uningested particles were quenched with trypan blue. In some experiments, the cells were treated with clone IM7 α–CD44 mAb. MFI of HA-microbeads inside B cells. Data derived from three independent assays. One-way ANOVA test was used in this graph, values are mean ± SD, (*$P<0.05$).

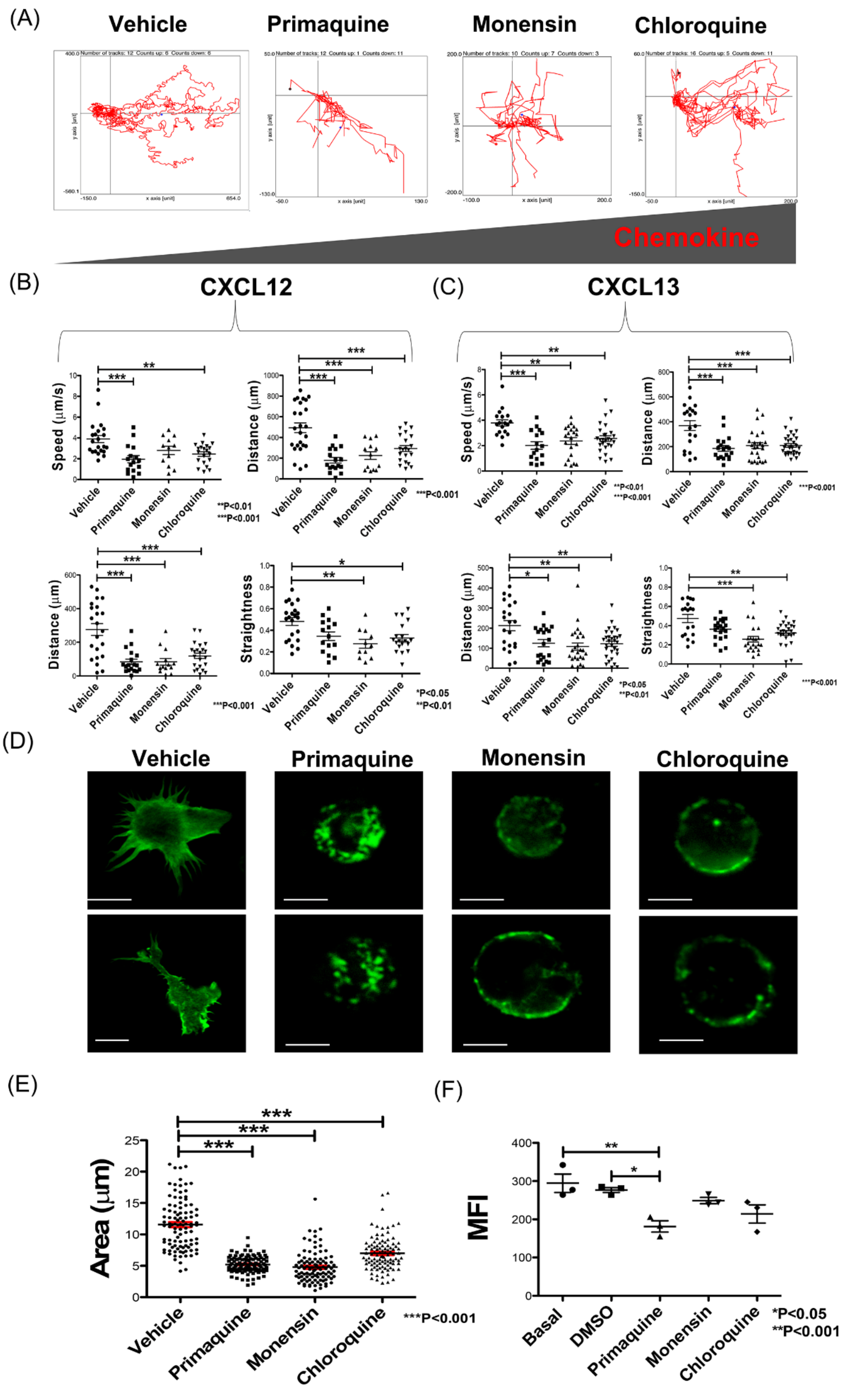

(A)
Vehicle
Primaquine
Monensin
Chloroquine
Chemokine
(B)
CXCL12
(C)
CXCL13
Speed (μm/s)
Distance (μm)
Straightness
Vehicle
Primaquine
Monensin
Chloroquine
**P<0.01
***P<0.001
*P<0.05
(D)
Vehicle
Primaquine
Monensin
Chloroquine
(E)
Area (μm)
***P<0.001
(F)
MFI
Basal
DMSO
Primaquine
Monensin
Chloroquine
*P<0.05
**P<0.001

Supplementary figure 12. Recycling process is crucial in several B lymphocytes phenomena. B-cells treated with vehicle, primaquine, chloroquine, monensin were placed on fibronectin-covered coverslips for 30 min, after that the cells were treated with CXCL13 or CXCL12 and then incubated for 20 min. The coverslips, with the cells attached, were gently washed with 2.0 mL PBS, and then, 100 μL of supplemented RPMI 1640 were pipetted onto the coverslips, which in turn were placed upside-down onto the Zigmond slides. One of the grooves in the Zigmond chamber was filled with supplemented medium (80 μL) and the chamber was placed under a microscope. A baseline image was obtained at 20x magnification and the other groove was then filled with the chemokine (50 ng), also dissolved in supplemented medium. Digital images of the cells were taken every 20 s for 30 min maintaining the temperature of the room at 35–39 °C. A) Trajectories of treated B lymphocytes were registered. Speed, accumulated- and Euclidian-distance, and straightness of treated B cells under CXCL12 (B) or CXCL13 (C) are shown. Confocal images of NIMR8-induced spreading of activated B cells treated with vehicle, primaquine, chloroquine or monensin are depicted in (D). The area of B lymphocytes in spreading is evaluated in (E). A total of 150 cells per data set, from three independent experiment, were analyzed. One-way ANOVA test was used in these experiments, values are mean ± SD (***P<0.001). Expression of CD44 in vehicle, primaquine-, chloroquine- or monensin-treated B cells (F). One-way ANOVA test was used in these experiments, values are mean ± SD, (n = 3)
